# Local Connections of Pyramidal Neurons to Parvalbumin-Producing Interneurons in Motor-Associated Cortical Areas of Mice

**DOI:** 10.1523/ENEURO.0567-20.2021

**Published:** 2022-02-02

**Authors:** Eriko Kuramoto, Yasuhiro R. Tanaka, Hiroyuki Hioki, Tetsuya Goto, Takeshi Kaneko

**Affiliations:** 1 Department of Oral Anatomy and Cell Biology, Graduate School of Medical and Dental Sciences, Kagoshima University, Kagoshima 890-8544, Japan; 2 Brain Science Institute, Tamagawa University, Tokyo 194-8610, Japan; 3 Department of Neuroanatomy, Juntendo University Graduate School of Medicine, Tokyo 113-8421, Japan; 4 Department of Morphological Brain Science, Graduate School of Medicine, Kyoto University, Kyoto 606-8501, Japan

**Keywords:** cerebral cortex, corticothalamic neurons, excitatory neurons, layer 6 neurons, motor area, parvalbumin-positive interneurons

## Abstract

Parvalbumin (PV)-producing neurons are the largest subpopulation of cortical GABAergic interneurons, which mediate lateral, feedforward, and feedback inhibition in local circuits and modulate the activity of pyramidal neurons. Clarifying the specific connectivity between pyramidal and PV neurons is essential for understanding the role of PV neurons in local circuits. In the present study, we visualized somas and dendrites of PV neurons using transgenic mice in which PV neurons specifically express membrane-targeted GFP, and intracellularly labeled local axons of 26 pyramidal neurons in layers 2–6 in acute slices of the motor-associated cortex from transgenic mice. We mapped morphologically distribution of inputs from a pyramidal neuron to PV neurons based on contact sites (appositions) between the axons from an intracellularly filled pyramidal neuron and the dendrites of PV neurons. Layer 6 corticothalamic (CT)-like pyramidal neurons formed appositions to PV neurons at a significantly higher rate than other pyramidal neurons. The percentage of apposed varicosities to all the labeled varicosities of layer 6 CT-like neurons was 28%, and that of the other pyramidal neurons was 12–19%. Layer 6 CT-like neurons preferentially formed appositions with PV neurons in layers 5b–6, while other pyramidal neurons uniformly formed appositions with PV neurons in all layers. Furthermore, both layer 6 CT-like and corticocortical-like neurons more frequently formed compound appositions, where two or more appositions were located on a dendritic branch, than other pyramidal neurons. Layer 6 CT neurons may contribute to intracortical information processing through preferential connections with PV neurons in layers 5b–6.

## Significance Statement

Local axons of 26 pyramidal neurons in layers 2–6 were intracellularly labeled in slices of the motor-associated cortex from transgenic mice in which somas/dendrites of parvalbumin (PV) neurons were labeled with GFP. We mapped the distribution of inputs to PV neurons based on contact sites (appositions) between axon varicosities of each pyramidal neuron and PV-neuron dendrites. The rate of appositions from layer 6 corticothalamic (CT)-like pyramidal neurons to PV neurons was higher than those of other pyramidal neurons, and connections were preferentially made in layers 5b–6. In contrast, other pyramidal neurons uniformly connected to PV neurons across all layers. Layer 6 CT neurons may contribute to intracortical information processing through preferential connections with PV neurons in layers 5b–6.

## Introduction

The laminar organization is a distinctive characteristic of the cerebral cortex, first delineated with cytoarchitecture and subsequently proven to correspond to gene expression and input-output selectivity (Hevner et al., 2003; Thomson and Bannister, 2003; Douglas and Martin, 2004; Molyneaux et al., 2007; Leone et al., 2008; Weiler et al., 2008; Shepherd, 2009; Watakabe, 2009). The principal excitatory neurons in each layer show cell-type-specific input-output connectivities and form local circuits that enable cortical computations (Kaneko, 2013; Shepherd and Yamawaki, 2021). The local cortical circuit contains 20–30% of inhibitory GABAergic interneurons, which are essential for stabilizing network dynamics (Tsodyks et al., 1997) and improving the efficiency and robustness of the neural code (Somers et al., 1995; Renart et al., 2010; Denève and Machens, 2016). Inhibitory GABAergic interneurons are divided into several subtypes according to the expression of peptides, receptors, and calcium-binding proteins (Xu et al., 2010; Rudy et al., 2011). Parvalbumin (PV)-producing interneurons are the largest subpopulation of cortical GABAergic interneurons (DeFelipe, 1993), and they display fast-spiking (FS) discharges (Kawaguchi and Kubota, 1993, 1998; Cauli et al., 1997). PV neurons may serve as mediators of lateral, feedforward, and feedback/recurrent inhibition (Kisvárday et al., 2000; Callaway, 2004; Lu et al., 2007), and contribute to the generation of γ rhythms (Tamás et al., 2000; Cardin et al., 2009; Sohal et al., 2009) in the cortical circuit, controlling the activity of pyramidal neurons. The function of a neural circuit is dictated by its connectivity. Recent studies have uncovered specific connectivity rules, such as preferential synaptic formation between specific neurons and the difference in the ratio of excitatory to inhibitory inputs between distal and proximal dendrites, leading to an understanding of the function of neural circuits (Motta et al., 2019; Karimi et al., 2020; Klinger et al., 2021). Thus, clarifying the specific connectivity rules between pyramidal and PV neurons is essential for understanding the role of PV neurons in local circuits.

Several methods have been used to investigate electrophysiological connections between pyramidal and PV neurons. Some studies have applied pair-recording or multi-recording using intracellular recording techniques to examine the electrophysiological properties of monosynaptic connections between two neurons. These methods have detected a strong connection from layer 6 corticothalamic (CT) pyramidal neurons to inhibitory interneurons. However, they have not fully revealed the subtypes of the interneurons (Thomson et al., 2002; Mercer et al., 2005; West et al., 2006). Other studies used a combination of whole-cell clamp recording and scanning laser photostimulation with caged-glutamate or transfected opsins, such as channelrhodopsin 2 in cortical slices (Dalva and Katz, 1994; Katz and Dalva, 1994). These techniques can stimulate a small number of neurons and have been applied to reveal connections from a group of excitatory neurons to a single PV neuron and have clarified that a group of pyramidal neurons in each layer most effectively drives individual PV neurons in the same or adjacent layers (Dantzker and Callaway, 2000; Xu and Callaway, 2009; Apicella et al., 2012; Bortone et al., 2014; Yamawaki and Shepherd, 2015). Conversely, however, it remains unknown how a single pyramidal neuron in each layer provides excitatory input to PV neurons.

Relatively few morphological studies have focused on the microcircuitry between pyramidal and inhibitory neurons. A previous electron-microscopic study has revealed that local axon collaterals of layer 6 CT neurons formed synapses more frequently with the dendritic shafts of presumed interneurons than with spiny dendrites of pyramidal neurons in layers 4–6 of the mouse somatosensory cortex (White and Keller, 1987). Although the previous result suggests the presence of strong connections from CT neurons to interneurons, subtypes of postsynaptic interneurons have not been identified.

To identify postsynaptic interneuron subtypes in the present study, we genetically visualized the information-receiving sites (somas and dendrites) of PV neurons using transgenic mice. Transgenic mice express a membrane-targeted green fluorescent protein (myrGFP-LDLRct) specifically at the information-receiving sites of PV neurons (Kameda et al., 2008, 2012). We performed intracellular recording/staining in acute slices of the motor-associated cortex from transgenic mice, and visualized axon collaterals of 26 pyramidal neurons. The inputs from a single pyramidal neuron in each layer to PV neurons were identified and quantified based on contact sites (appositions) between the axon varicosities of a single pyramidal neuron and the somas and dendrites of PV neurons. This method allows us to compare how individual pyramidal neurons in each layer input to PV neurons and provides morphological evidence for the existence of preferential connections from layer 6 CT-like pyramidal neurons to PV neurons.

## Materials and Methods

### Animals

All procedures involving animals were performed in accordance with the National Institutes of Health *Guide for the Care and Use of Laboratory Animals*. Mice were maintained under a 12/12 h light/dark cycle and provided access to food and water *ad libitum*. The present experiments were approved by the Committees for Animal Care (Med Kyo 12013, 12014) and the Recombinant DNA Study (120093) at Kyoto University. Seventy-nine transgenic mice (51 males and 28 females, three to six months old, 20–36 g body weight; Kameda et al., 2012) were used in the present study. The transgenic mice express membrane-targeted GFP (myrGFP-LDLRct) specifically in the somas and dendrites of PV neurons (PV/myrGFP-LDLRct mice). The dendrites were clearly observed with GFP immunoreactivity up to their ends, and small swellings were usually found ∼1.0 μm from the end of the dendrites (Kameda et al., 2012; see their Figs. 2*A–C”*, 7*A*). All efforts were made to minimize animal suffering and reduce the number of animals used in the present study.

### Preparation of the cortical slice, intracellular recording, and fixation

**Figure 1.**
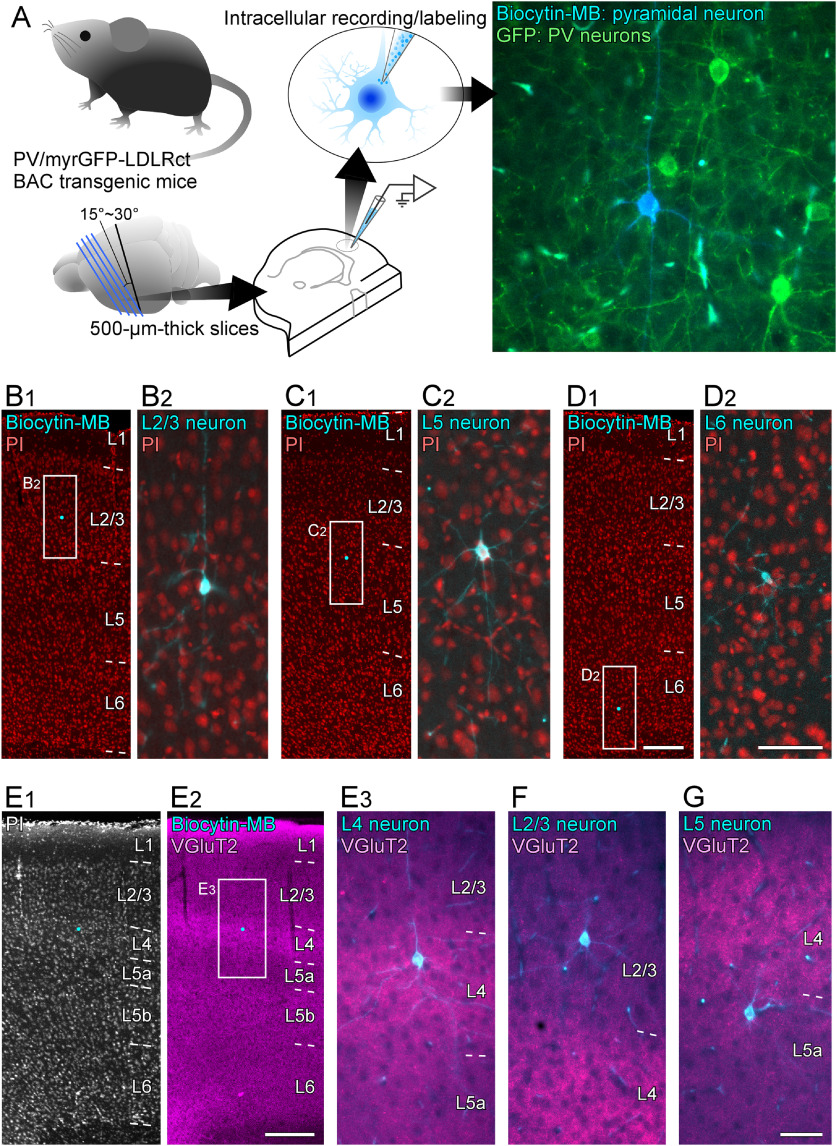
Intracellular recording/labeling of pyramidal neurons and characterization of labeled pyramidal neurons. Schematic of analysis for input maps from single pyramidal neuron to PV neurons using a combination of intracellular recording/labeling technique for single pyramidal neuron (biocytin-marina blue; biocytin-MB) and genetic labeling technique for visualization of dendrites and somas of PV neurons (bacterial artificial chromosome transgenic mice expressing the somatodendritic membrane-targeted green fluorescent protein in PV neurons, PV/myrGFP-LDLRct BAC transgenic mice; ***A***). Identification of intracellularly labeled pyramidal neurons in reference to Nissl-like staining of propidium iodide (PI; ***B1–E1***) and immunoreactivity for VGluT2 (***E2–G***). Small cyan dots in ***B1***, ***C1***, ***D1***, ***E1*, *E2*** indicate the location of biocytin-labeled pyramidal neuronal cell bodies. The frontal sections which contained biocytin-labeled neuronal cell bodies were stained with PI and marina blue (MB)-conjugated streptavidin, and observed under the fluorescent microscope (***B1–E1***). The sections were further immunostained for VGluT2 (***E2–G***). Extended Data Figure 1-1 shows the localization of all the 26 analyzed pyramidal neurons. Scale bars: 200 μm (in ***D1***; applies to ***B1***, ***C1***, ***D1***), 50 μm (in ***D2***; applies to ***B2***, ***C2***, ***D2***), 200 μm (in ***E2***; applies to ***E1***, ***E2***), and 50 μm (in ***G***; applies to ***E3***, ***F***, ***G***).

Seventy transgenic mice (42 males and 28 females) were deeply anesthetized by isoflurane inhalation and decapitated. The slices were prepared as follows. Briefly, to retain neuronal viability in the slices, we used an *N*-methyl-d-glucamine-based cutting solution (Tanaka et al., 2008, 2011a,b), containing 147 mm *N*-methyl-d-glucamine, 20 mm HEPES, 1 mm KCl, 1.3 mm KH_2_PO_4_, 2.5 mm MgSO_4_, 1 mm CaCl_2_, and 10 mm d-glucose (pH was adjusted to 7.4 by HCl). The brains were quickly removed and cut into 500-μm-thick slices in the cutting solution saturated with 95% O_2_ and 5% CO_2_ gas on a microslicer (Dosaka EM). The cutting direction was optimized in the preliminary experiments to be parallel to the apical dendrites of pyramidal neurons in the motor-associated areas (Fig. 1*A*). The slices were preincubated at 20°C for 1–8 h in artificial cerebrospinal fluid (ACSF) saturated with 95% O_2_ and 5% CO_2_ gas. The ACSF was composed of 124 mm NaCl, 3.3 mm KCl, 26 mm NaHCO_3_, 1.3 mm KH_2_PO_4_, 2.5 mm CaCl_2_, 1 mm MgSO_4_, and 10 mm D-glucose (pH 7.4, when saturated with 95% O_2_ and 5% CO_2_ gas). The slices were then placed in an interface chamber system (Model BSC-BU with BSC-HT attachment; Warner Instruments) at 34–35°C and perfused with the ACSF. Glass micropipettes were made with a puller (P-97; Sutter) and filled with 3% (w/v) biocytin (Sigma-Aldrich) dissolved in 2 m potassium methylsulfate and 50 mm Tris-HCl, pH 7.4. The resistance of the sharp electrode was 100–250 MΩ. To maximize the morphological recovery of neuronal processes, we recorded neurons in the middle two-fifths of the slice thickness (150–350 μm from the slice cut surface) in the cortical slices. After impalement, the response of the pyramidal neuron to current injection was recorded using a high-input impedance DC amplifier with an active bridge circuit (IR-183, Neurodata) and stored in a computer through an analog-digital converter (PowerLab, AD Instruments). Before releasing the impaled neuron, biocytin was injected by passing 200-ms-long, 0.1- to 0.5-nA positive pulses at 2.0 Hz. At the end of the recording, the slices were fixed in 3% formaldehyde, 0.003% glutaraldehyde, and 75%-saturated picric acid in 0.1 m sodium phosphate, pH 7.4 at 25°C for 20 h.

10.1523/ENEURO.0567-20.2021.f1-1 Extended Data Figure 1-1 The somal locations of intracellularly labeled pyramidal neurons in the present study. The somal locations were projected onto the nearest frontal plane of Nissl-stained and VGlu-stained sections and serially numbered from the superficial layer to the deep layer, from the rostral to caudal, and from the medial to lateral portions of the motor-associated cortical areas. The horizontal broken lines indicate the border of cortical layers, and the vertical broken lines indicate the border of cortical areas, which were determined in the Nissl-stained sections with the aid of VGlu immunoreactivity in the adjacent sections. Yellow, green, peal-blue, blue, and purple marks indicate somal locations of layer (L)2/3, L4, L5a, L5b, and L6 pyramidal neurons, respectively. The purple-filled circles and rectangles indicate layer 6 CC-like and CT-like pyramidal neurons, respectively. FL, forelimb region of the primary somatosensory motor area; HL, hindlimb region of the primary somatosensory motor area; M1, the primary motor area; M2, the secondary motor area. Scale bars: 1 mm (in ***A6***; applies to ***A1****–****A6***) and 500 μm (in ***D6***; applies to ***B1****–****D6***). Download Figure 1-1, TIF file.

### Visualization of recorded neurons and GFP-positive PV neurons

After cryoprotection with 30% sucrose in 10 mm phosphate-buffered 0.85% saline (PBS), the slices were further cut frontally into 30-μm-thick sections on a freezing microtome. In the following processes, each section was separately incubated in a well at 22–26°C, and the incubation was followed by rinsing with PBS containing 0.3% Triton X-100 and 0.01% ProClin 950 (46885-U; Merck Millipore; PBS-X). The sections were first soaked for 30 min in 1% H_2_O_2_ in PBS to suppress endogenous peroxidase activity, and the sections were then incubated with 5 μg/ml marina blue-conjugated streptavidin (S-11221; Thermo Fisher Scientific) for 1 h. The sections were then observed under an epifluorescence microscope (Axiophot; Zeiss) with the filter sets for marina blue (359–371 and 397–490 nm for excitation and emission, respectively) to locate the biocytin-injected neuronal cell bodies. The sections were then incubated overnight with 0.1 μg/ml affinity-purified anti-GFP rabbit antibody (Tamamaki et al., 2000; Nakamura et al., 2008) in PBS-X containing 0.12% λ-carrageenan (035-09693; Wako Chemicals), 0.02% NaN_3_, and 1% normal goat serum (S-1000; Vector Laboratories; PBS-XCG). The sections containing biocytin-injected neuronal cell bodies were incubated overnight with 2 μg/ml affinity-purified anti-vesicular glutamate transporter 2 (VGluT2) guinea pig antibody (Fujiyama et al., 2001), and then for 2 h with 10 μg/ml Alexa Fluor 594-conjugated anti-(guinea pig IgG) goat antibody (A-11 076; Thermo Fisher Scientific) in PBS-XCG. Under an epifluorescence microscope (Axiophot; Zeiss), VGluT2 immunoreactivity (Alexa Fluor 594) was detected under 530- to 585-nm excitation and ≥615-nm emission conditions. The sections were incubated further for 2 h with 10 μg/ml propidium iodide in PBS-X, and the location of the labeled neurons was re-examined in reference to Nissl-like staining (propidium iodide, excitation 530–585 nm, emission ≥615 nm).

All sections were incubated for 1 h with ABC-Elite (1:50; Vector Laboratories) in PBS-X, and peroxidase in the ABC-Elite bound to intracellularly injected biocytin was then developed in blue-black by incubation for 30–60 min with 0.02% (w/v) diaminobenzidine-4HCl (DAB; 347-00904, Dojindo), 10 mm nickel ammonium sulfate and 0.0001% (v/v) H_2_O_2_ in 50 mm Tris-HCl, pH 7.6. After a 15 min incubation with 2% (w/v) NaN_3_ in PBS to inactivate the peroxidase in the ABC-Elite, the sections were further incubated for 1 h with 10 μg/ml biotinylated anti-(rabbit IgG) goat antibody (BA-1000, Vector Laboratories), and subsequently for 1 h with ABC-Elite (1:50) in PBS-X. The peroxidase in GFP-labeled neurons was developed in pink by incubation for 30–45 min with 0.1% (w/v) Tris-aminophenylmethane (TAPM; Nacalai Tesque), 0.07% (v/v) *p*-cresol (C85751; Sigma-Aldrich), and 0.002% (v/v) H_2_O_2_ in 50 mm Tris-HCl, pH 7.6 (Kaneko et al., 1994). The sections were mounted on gelatin-coated glass slides, dried, washed in running water for 2 min, dried again overnight, cleared in xylene, and coverslipped.

The cytoarchitectonic areas and layers were determined in Nissl-stained and VGluT2-immunostained sections of slices adjacent to the slices used for intracellular injection, according to Swanson (2004), Paxinos and Watson (2007), and Paxinos and Franklin (2019) with reference to Donoghue and Wise (1982) and Zilles (1985). The primary (M1) and secondary motor areas (M2) corresponded to the lateral and medial agranular fields of Donoghue and Wise (1982), whereas the hindlimb (HL) and forelimb areas (FL) were granular fields, which were often included in the primary somatosensory area. However, because the HL and a medial part of the FL have been reported to share the electrophysiological and morphological characteristics of the M1 (Hall and Lindholm, 1974; Donoghue et al., 1979; Donoghue and Wise, 1982), the motor-associated areas in the rodent cerebral cortex here include the M1, M2, HL, and FL areas. Layer 1, layer 2/3, layer 5, and layer 6 can be identified by cytoarchitecture. Layer 2/3 and layer 5 were further subdivided by immunoreactivity for VGluT2; lower parts of layer 2/3 and layer 5 showed more intense immunoreactivity for VGluT2 than the upper parts of layer 2/3 and layer 5, respectively (Cho et al., 2004a; Ueta et al., 2013; Kawaguchi, 2017). Since VGluT2 is considered to be located in the thalamocortical axon terminals in the cerebral cortex (for review, see Fujiyama et al., 2001; Kaneko and Fujiyama, 2002; Fremeau et al., 2004), the lower parts of layer 2/3 and layer 5 would receive thalamic inputs intensely and might be functionally different from the upper parts of layer 2/3 and layer 5, respectively (Cho et al., 2004a; Ueta et al., 2013; Kawaguchi, 2017). In the present study, the upper and lower parts of layer 2/3 were named layer 2/3 and layer 4, respectively, according to Cho et al. (2004a), and the upper and lower parts of layer 5 were called layer 5a and layer 5b, respectively, according to Ueta et al. (2013) and Kawaguchi (2017).

### Immunoelectron microscopy

A solution of 3% biocytin dissolved in 2 m potassium methylsulfate and 50 mm Tris-HCl (pH 7.4) was used to label the axon collaterals of cortical neurons. Six male PV/myrGFP-LDLRct transgenic mice were anesthetized with sodium pentobarbital (60 mg/kg, i.p.), and the 3% biocytin solution was electro-osmotically delivered into the motor-associated cortical areas (1.0 mm anterior to the bregma, 1.5 mm lateral to the midline, and 0.8 mm deep from the brain surface) by passing positive 2-μA current pulses at 7-s intervals through a glass micropipette for 20 min. The six biocytin-injected mice were killed 48 h after the injection. Briefly, the mice were deeply anesthetized again with sodium pentobarbital (100 mg/kg, i.p.) and perfused transcardially with 10 ml of PBS, and then perfused with 200 ml of 4% paraformaldehyde (26126-25; Nacalai Tesque) and 0.05% glutaraldehyde (17003-92; Nacalai Tesque) in 0.1 m sodium phosphate buffer (PB; pH 7.4). After postfixation for 4 h with 4% paraformaldehyde in 0.1 m PB at 4°C, the brain blocks were cut into 50-μm-thick frontal sections with a vibratome (Microslicer DTK-1000; Dosaka). Endogenous peroxidase activity in the vibratome sections was suppressed by incubation with 1% H_2_O_2_ in PBS for 30 min at 22–26°C, and the sections were thoroughly washed with PBS at 22–26°C and preincubated in PBS containing 20% (v/v) normal goat serum (S-1000; Vector Laboratories) for 1 h at 22–26°C to block nonspecific binding of antibodies. The following incubations were conducted at 4°C in PBS containing 10% normal goat serum and 0.2% Photoflo (FUJI-FILM) unless otherwise stated. After overnight incubation with a mixture of ABC-Elite (1:50) and 0.1 μg/ml anti-GFP rabbit IgG, the bound ABC was visualized by peroxidase reaction in the DAB/nickel reaction mixture. After the inactivation of peroxidase in ABC with NaN_3_, the sections were incubated for 4 h with 10 μg/ml biotinylated anti-(rabbit IgG) goat antibody and subsequently for 4 h with ABC-Elite (1:50). The bound ABC in the sections was developed in brown for 20–40 min at 22–26°C in a DAB reaction mixture containing 0.02% DAB and 0.001% H_2_O_2_ in 50 mm Tris-HCl (pH 7.6). The sections were postfixed with 1% osmium tetroxide (25727-01; Nacalai Tesque) in 0.1 m PB, stained with 1% uranyl acetate (8473; Merck Millipore) in distilled water, dehydrated in ethanol series, and flat-embedded in epoxy-resin (Luveak-812, Nacalai Tesque). Once the resin was polymerized, the tissue samples were cut into ultrathin sections (70 nm) using an ultramicrotome Reichert-Nissei Ultracut S (Leica). The ultrathin sections were mounted on grids and examined under an electron microscope H7650 (Hitachi).

### Morphological reconstruction of single excitatory neurons and analysis of close appositions between their axon varicosities and PV-neuron dendrites

The cell bodies, dendrites, and axon collaterals of intracellularly stained pyramidal neurons were reconstructed as follows. The cortical motor-associated areas of the frontal sections were automatically captured into a large color image with a spatial resolution of 0.258 μm/pixel using a digital slide scanner TOCO (CLARO). On the images, we traced and digitized the axon fibers with a pen tablet (Bamboo Tablet; Wacom) and CANVAS X software (ACD Systems International Inc.). The axon fibers were thereby reconstructed two-dimensionally to a collection of many short Bézier curves section by section onto a frontal plane, and the digitized fibers from all the sections were superimposed on the computer. The length and color code of the Bézier curves in a Canvas file were automatically determined curve by curve and written into an Excel file using an AppleScript macro. The length of the two-dimensionally reconstructed axon fibers was shorter than the actual length of the axons, which were distributed in three dimensions. Previously, we estimated the length of axons in three dimensions by multiplying the length of the two-dimensionally reconstructed axons by π/2 ≒ 1.5708 (Cho et al., 2004a,b; Kuramoto et al., 2009, 2015, 2017a,b; Matsuda et al., 2009; Tanaka et al., 2011b; Ohno et al., 2012; Nakamura et al., 2015). However, we noticed that “4/π ≒ 1.2732” had to be used instead of “π/2 ≒ 1.5708” for the correct estimation (Baddeley and Jensen, 2004; see their Eq. 2.38). Therefore, in the present study, we estimated the length of axons in three dimensions by multiplying the length of the two-dimensionally reconstructed axons by 4/π.

After the two-dimensional reconstruction of neuronal processes, varicosities on the axon collaterals of intracellularly labeled neurons were plotted on the Bézier curves under a microscope with an oil-immersion 100× objective lens (PlanApo100; numerical aperture = 1.4; Olympus). When axon varicosities were ≥1.5-fold thicker than intervaricose fibers, they were presumed to be presynaptic axon varicosities (Kuramoto et al., 2009, 2015; Ohno et al., 2012; Nakamura et al., 2015). During the plotting of the axon varicosities, we carefully examined whether the axon varicosities were closely apposed to the dendritic processes or cell bodies of PV neurons, while frequently changing the microscopic focus with the 100× objective lens. To measure fine morphological indices, such as diameter and area of axon varicosities, digital images were captured under a microscope with a 100× objective lens and a DP25 camera (Olympus), and analyzed using CANVAS X software. For statistical analysis, one-way or two-way ANOVA, Tukey’s test and two-tailed *t* test, GraphPad Prism 9 (GraphPad Software Inc.) and Excel (Microsoft) were used.

### Input maps from pyramidal neurons to PV neurons

If synapse formation is random, the number of synapses is directly proportional to the number of axon varicosities and the density of postsynaptic targets (dendrites and cell bodies) in the region of interest. This logic is known as Peters’ rule (Peters and Feldman, 1976; Peters and Payne, 1993; Binzegger et al., 2004). If a presynaptic neuron *j* with a number ( $S_{j}^{u}$) of axon terminals (presynaptic sites) uniformly and randomly forms synapses with postsynaptic neurons in cortical layer *u*, the number of synapses $S_{ji}^{u}$ between the presynaptic neuron *j* and postsynaptic neurons of *type i* can be estimated using the following formula:

(1) $$S_{ji}^{u}=S_{j}^{u}\cdot \frac{N_{i}^{u}}{N_{}^{u}}.$$

Here, *N^u^* is the number of all neurons in layer *u*, and $N_{i}^{u}$ is the number of postsynaptic neurons of *type i*. Therefore, $\frac{N_{i}^{u}}{N^{u}}$ is the proportion of *type i* neurons among all neurons in layer *u*. In the scope of our study, the postsynaptic neurons of *type i* are PV neurons, and the presynaptic neuron *j* represents each pyramidal neuron.

To fit formula (1) to the present experiments, we approximated the presynaptic sites $S_{j}^{u}$ and the synapses $S_{ji}^{u}$ with the varicosities of each pyramidal (pyr) neuron $V_{pyr}^{u}$ and close appositions $C_{pyr-PV}^{u}$, respectively. Furthermore, we used the area proportion of PV-neuron dendrites in layer *u* $A_{PV}^{u}$ instead of $\frac{N_{i}^{u}}{N^{u}}$ since most of the closely apposed varicosities of pyramidal neurons were formed with PV-neuron dendrites (see Results). Thus, we focused on the analysis of close appositions with PV-neuron dendrites in the present study, and from formula (1), we deduced a relationship:

(2) $$C_{pyr-PV}^{u}\propto V_{pyr}^{u}\cdot A_{PV}^{u}.$$

If the formation of close appositions (potential synapses) from pyramidal neurons to PV-neuron dendrites is entirely random and if the surface area of the presynaptic structure is infinitely small, the proportional constant of Equation 2 should ideally be 1 for all pyramidal neuron types of all layers. However, it is actual that axon varicosities have sizes of ∼ 1 μm and that we counted close appositions $C_{pyr-PV}^{u}$ when any portion of the varicosities contacted to the PV-neuron dendrites. In this case, the proportional constant should be slightly larger than 1, even in the random case. To assess the variability of the proportionality among pyramidal neuron types and layers, we calculated the proportional constant κ from the actual data of reconstructed pyramidal neurons in each layer using Equation 3:

(3) $$\kappa =\frac{C_{pyr-PV}^{u}}{V_{pyr}^{u}}\cdot \frac{1}{A_{PV}^{u}}.$$

The parameter, $\frac{C_{pyr-PV}^{u}}{V_{pyr}^{u}}$ was obtained as the proportion of appositions within layer *u* of each pyramidal neuron and $A_{PV}^{u}$ was obtained as described in the following section. Since the size of axon varicosities did not show apparent variations across pyramidal neuron types or layers where axon arbors had spread (see Table 1 varicosity size), κ allows us to compare the preference to PV neurons among pyramidal neuron types or layers.

### Measurement of area proportion of PV-neuron dendrites

To measure the area proportion of PV-neuron dendrites in cortical layers, frontal 30-μm-thick sections obtained from three male PV/myrGFP-LDLRct mice were immunostained as follows. Briefly, sections were incubated overnight with a mixture of 2 μg/ml anti-VGluT2 guinea pig antibody in PBS-XCG. After several washes with PBS-X, the sections were incubated for 4 h with 10 μg/ml Alexa Fluor 594-conjugated anti-(guinea pig IgG) goat antibody (A-11 076; Thermo Fisher Scientific) and 10 μg/ml 4’,6-diamino-2-phenylindole (DAPI) in PBS-XCG. The sections were then washed thoroughly in PBS, mounted on APS-coated slide glasses (APS-01; Matsunami Glass Ind), air dried, and coverslipped with 90% (v/v) glycerol and 2.5% (w/v) triethylendiamine (antifading agent) in 20 mm Tris-HCl, pH 7.6. The sections were observed under an epifluorescence microscope (Axiophot; Zeiss) with appropriate filter sets for DAPI (excitation 359–371 nm, emission 397–490 nm), GFP (excitation 450–490 nm, emission 514–565 nm), and Alexa Fluor 594 (excitation 530–585 nm, emission ≥615 nm), or a confocal laser-scanning microscope (LSM 700; Zeiss) with appropriate sets of laser beams and emission windows for DAPI (excitation 405 nm, emission 414–515 nm), GFP (excitation 488 nm, emission 505–530 nm), and Alexa Fluor 594 (excitation 594 nm, emission 630–800 nm). To measure the area proportion of PV (GFP)-positive dendrites in the motor-associated areas, digital images were captured under a confocal laser-scanning microscope (LSM 700; Zeiss) with an oil-immersion 63× objective lens (Plan Apochromat, numerical aperture = 1.4; Zeiss), pinhole of 1.0 Airy unit, and zoom factor of 10. We selected six sections, which were spaced at regular intervals within the motor-associated areas per mouse, and captured one image each in the M1, M2, FL, and HL areas per section. Thus, we captured 18-images for each area from three mice in total. The digital images were processed and analyzed using the ImageJ software (version 1.48; National Institutes of Health).

## Results

### Localization of intracellularly labeled pyramidal neurons

In the present study, we used sharp electrodes to impale pyramidal neurons in the motor-associated areas (M1, M2, FL, and HL) in 500-μm-thick cortical slices of the PV/myrGFP-LDLRct mice, in which PV neurons specifically expressed somatic/dendritic membrane-targeted GFP (Fig. 1*A*). Fifty-five pyramidal neurons in 50 slices from 31 mice (20 males and 11 females) were intracellularly recorded and labeled with biocytin, and their locations were identified based on cytoarchitecture (Fig. 1*B–D*) and on thalamocortical inputs with chemoarchitecture of VGluT2 immunoreactivity (Fig. 1*E–G*). Of all neurons labeled, 10, 5, 8, 17, and 15 pyramidal neurons were located in layer 2/3, layer 4, layer 5a, layer 5b, and layer 6, respectively.

The 55 intracellularly biocytin-labeled neurons were stained with ABC-DAB to visualize their cell bodies, dendrites, and axon arbors. All the 55 neurons were pyramidal neurons (Lorente de Nó, 1938), except for two inverted pyramidal neurons located in layer 6 (Mendizabal-Zubiaga et al., 2007; Thomson, 2010). Twenty-nine of the 55 neurons were not processed further, mainly because their axon labeling faded away at the distal sites of their axon arbors. Thus, five, five, three, three, and 10 pyramidal neurons in layer 2/3, layer 4, layer 5a, layer 5b, and layer 6, respectively (26 neurons in total), were further processed, and numbered according to the location of their cell bodies in the motor-associated areas from the superficial layer to deep layer, from the rostral to caudal and from the medial to lateral portions (Extended Data Fig. 1-1).

### Distribution of axon collateral arbors and appositions between axon varicosities of a pyramidal neuron and somas/dendrites of PV neurons

**Figure 2.**
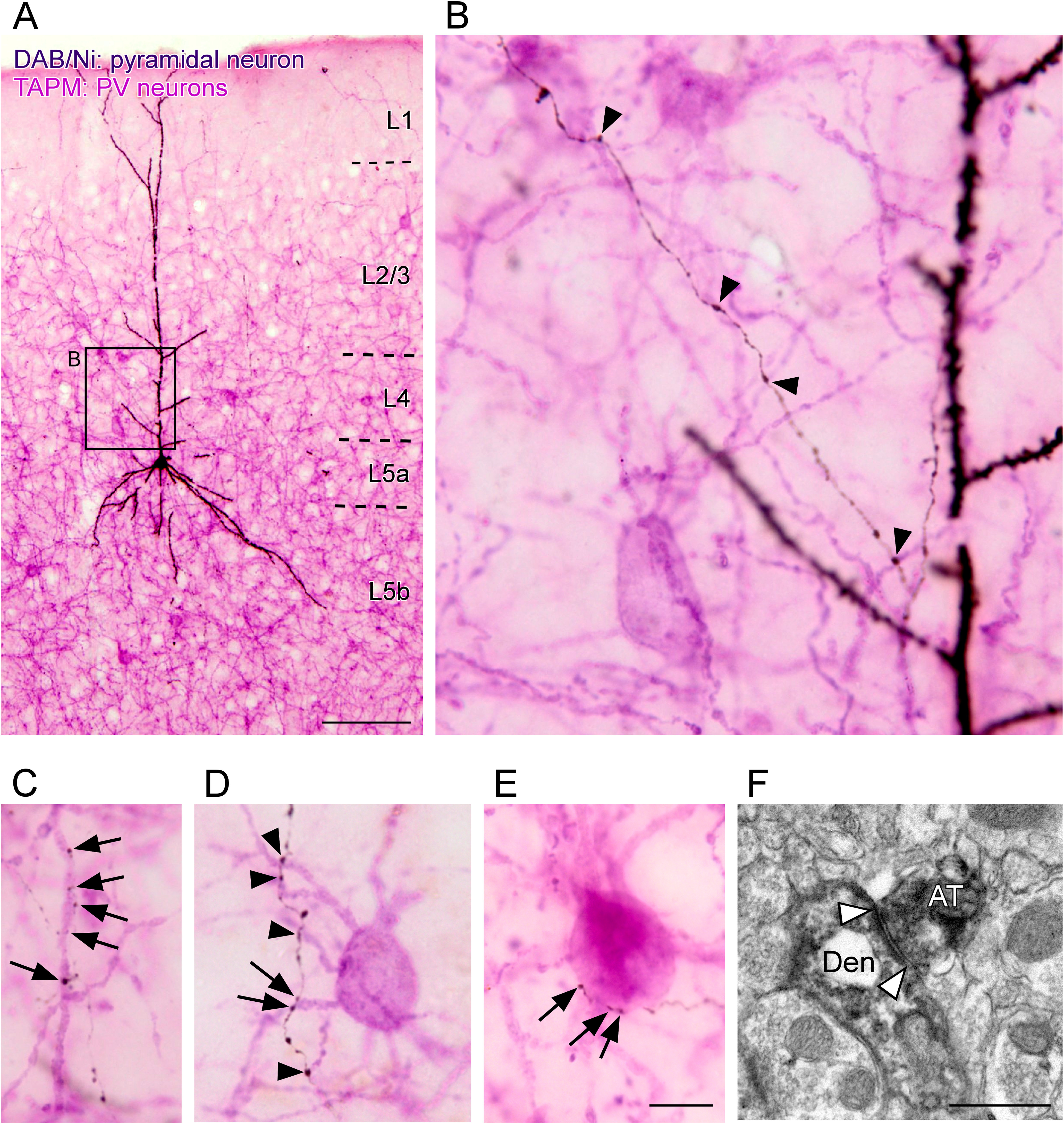
Light and electron microscopic findings of close appositions formed between the axon varicosities of pyramidal neurons and somas/dendrites of PV neurons. A biocytin-labeled pyramidal neuron was developed in blue-black with the DAB/nickel reaction, whereas almost all the somas and dendrites of PV neurons were visualized in pink by immunostaining for GFP with the TAPM/p-cresol reaction (***A***). Arrows and arrowheads in ***B–E*** indicate the axon varicosities, which were closely apposed to dendrites (***B–D***) and the cell body (***E***) of PV neurons. Some apposed varicosities formed multiple appositions to a dendritic branch or a soma; for example, five, two, and three varicosities closely apposed to a dendritic branch and a soma (arrows in ***C***, ***D***, ***E***, respectively). White arrowheads in ***F*** indicate a typical asymmetric synaptic contact that was made between the biocytin-labeled axon terminal (AT) and the dendrite (Den) with GFP immunoreactivity. Biocytin and GFP were visualized with the DAB/nickel and DAB reactions, respectively. Extended Data Figure 2-1 shows other examples of asymmetric synaptic contact. Scale bars: 100 μm (***A***), 10 μm (in ***E***; applies to ***B–E***), and 500 nm (***F***).

In addition to axon arbors of pyramidal neurons with varicosities (putative presynaptic structures, White et al., 2004; De Paola et al., 2006; Gala et al., 2017), dendrites of GFP-positive PV neurons were visualized using TAPM (Fig. 2*A*, pink; Kaneko et al., 1994). This double visualization enabled us to anatomically examine the inputs from a pyramidal neuron to PV neurons. Some axon varicosities of the pyramidal neurons were closely apposed to the dendrites or in a few cases, the cell bodies of PV neurons (hereafter called “apposed varicosities”; Fig. 2*B–E*, arrowheads and arrows). In the specimens prepared for electron microscopy (for details, see Materials and Methods), we randomly chose 30 varicosities apposed to dendrites and 10 varicosities apposed to cell bodies (40 apposed varicosities in total) and checked whether these varicosities made synaptic contacts with GFP-immunopositive postsynaptic structures. All the apposed varicosities examined were confirmed as presynaptic structures electron-microscopically. Of these, 29 were in synaptic contacts with GFP-immunopositive postsynaptic profiles. Of the 29 synaptic contacts, 22 were with dendrites, and the other seven were with cell bodies (Fig. 2*F*; Extended Data Fig. 2-1). The rate (22/30 = 73% for dendrites; 7/10 = 70% for cell bodies) is consistent with previous reports (Markram et al., 1997; Kaneko et al., 2000; Cho et al., 2004b; Helmstaedter et al., 2008; Kameda et al., 2012). The other 11 apposed varicosities, which appeared closely located to somas/dendrites of PV neurons under light microscopy, were proved to be presynaptic structures with GFP-negative postsynaptic targets.

10.1523/ENEURO.0567-20.2021.f2-1 Extended Data Figure 2-1 Close appositions found under a light microscope were examined using electron microscopy. GFP and biocytin were visualized as brown and blue-black with the DAB and DAB/nickel reactions, respectively (***A***, ***G***). Black arrowheads in ***A***, ***B***, ***G–I*** indicate close appositions. Axosomatic (***A***, ***B***) and axodendritic (***G–I***) appositions were found to form asymmetrical synapses (***C***, ***J***, respectively). White arrowheads in ***C***, ***E***, ***F***, ***J–P*** indicate the typical asymmetric synaptic contacts that were made between the biocytin-labeled axon terminals (AT) and the cell bodies (CB) or the dendrites (Den) with GFP immunoreactivity. Scale bars: 10 μm (***A***), 10 μm (***B***), 1 μm (***C***), 5 μm (***D***), 10 μm (in ***H***; applies to ***G***, ***H***), 5 μm (***I***), 1 μm (***J***), and 500 nm (in ***P***; applies to ***E*, *F*, *K–P***). Download Figure 2-1, TIF file.

**Figure 3.**
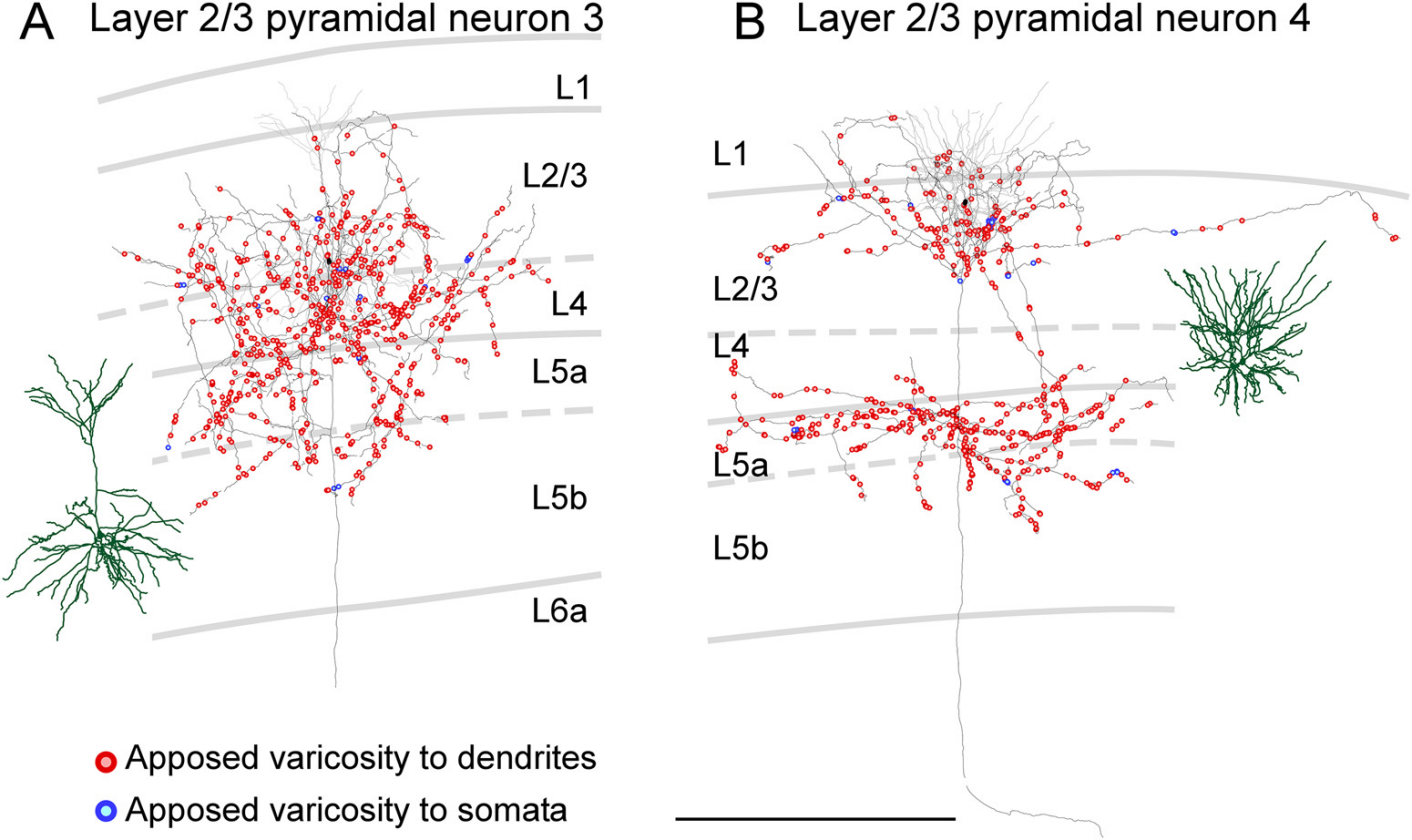
Distribution of axon varicosities of layer 2/3 pyramidal neurons in close appositions to PV neurons. Two representative layer 2/3 pyramidal neurons are shown (***A***, ***B***). Axons of pyramidal neurons were two-dimensionally reconstructed and projected onto the frontal plane. Black lines and filled circles represent axons and cell bodies of pyramidal neurons, respectively; red and blue circles indicate the axodendritic and axosomatic appositions, respectively. One each apposed varicosity is represented with one red or blue circle. Dark green and gray lines indicate reconstructed dendrites and their actual positions in the cortical layers, respectively. Recently, layer 2/3 pyramidal neurons are suggested to be divided into layer 2 and layer 3 neurons based on the morphology of their dendrites. In layer 2 neurons, the horizontal span of the apical dendrites is larger than that of the basal dendrites, whereas, in layer 3 neurons, the horizontal span of the basal dendrites is larger. According to this criterion, neuron 4 (***B***) shown here is presumed to be layer 2 neurons because the horizontal span of their apical dendrites is larger than that of basal dendrites. On the other hand, neuron 3 (***A***) shown here is assumed to be layer 3 neurons because the horizontal span of their basal dendrites is larger than that of apical dendrites. The other layer 2/3 pyramidal neurons are shown in Extended Data Figure 3-1. Scale bar: 500 μm.

Next, we reconstructed the dendrites and axon collateral arbors of the labeled pyramidal neurons and plotted axon varicosities, while examining whether each varicosity was apposed to the dendritic processes or cell bodies of PV neurons. Four of the five pyramidal neurons in layer 2/3 had a developed apical dendritic shaft (neurons 1–3 and 5 in Fig. 3; Extended Data Fig. 3-1); however, the remaining one pyramidal neuron possessed an undeveloped one (neuron 4 in Fig. 3*B*) like “modified pyramids” of O’Leary (1941). The main axons of pyramidal neurons in layer 2/3 projected toward the white matter and emitted many axon collaterals along its course. The longest average length of axon collaterals per pyramidal neuron was observed for pyramidal neurons in layer 2/3 (Table 1). Pyramidal neurons in layer 2/3 were classified into two groups in terms of axon collateral arbors: (1) neurons with one axon collateral cluster in layers 1–4 (neurons 1–3; Fig. 3; Extended Data Fig. 3-1) and (2) neurons with two clusters; one cluster in layers 1–4 and another one in layers 5–6 (neurons 4 and 5; Fig. 3; Extended Data Fig. 3-1). As expected from the axon collateral distributions, the ratio of the number of axon varicosities in layers 5–6 to that in layers 1–4 was 0.14–0.41 (0.26, mean) for the former neurons and it was surpassed by 0.54–0.71 (0.63) for the latter neurons. Some axon varicosities of pyramidal neurons in layer 2/3 were closely apposed to the dendrites of PV neurons in both layers 1–4 and layers 5–6 (Fig. 3; Extended Data Fig. 3-1). The mean percentage of apposed varicosities to all the labeled varicosities of pyramidal neurons in layer 2/3 with two axon collateral clusters was 11.1% and that of pyramidal neurons in layer 2/3 with one cluster was 13.3%, suggesting that these two types similarly contributed to inputs on PV neurons at least in number.

10.1523/ENEURO.0567-20.2021.f3-1 Extended Data Figure 3-1 Distribution of axon varicosities of layer 2/3 pyramidal neurons in close appositions to PV neurons. Axons of layer 2/3 pyramidal neurons were reconstructed two-dimensionally and projected onto the frontal plane. Black lines and filled circles represent axons and cell bodies of pyramidal neurons, respectively; red and blue circles indicate the axodendritic and axosomatic appositions, respectively. Each apposed varicosity is represented by a red or blue circle. Dark green and gray lines indicate reconstructed dendrites and their actual positions in the cortical layers, respectively. Recently, layer 2/3 pyramidal neurons are suggested to be divided into layer 2 and layer 3 neurons based on the morphology of their dendrites. In layer 2 neurons, the horizontal span of the apical dendrites is larger than that of the basal dendrites, whereas in layer 3 neurons, the horizontal span of the basal dendrites is larger. According to this criterion, the layer 2/3 neurons shown here are presumed to be layer 3 neurons because the horizontal span of their basal dendrites is larger than that of apical dendrites. Scale bar: 500 μm. Download Figure 3-1, TIF file.

**Figure 4.**
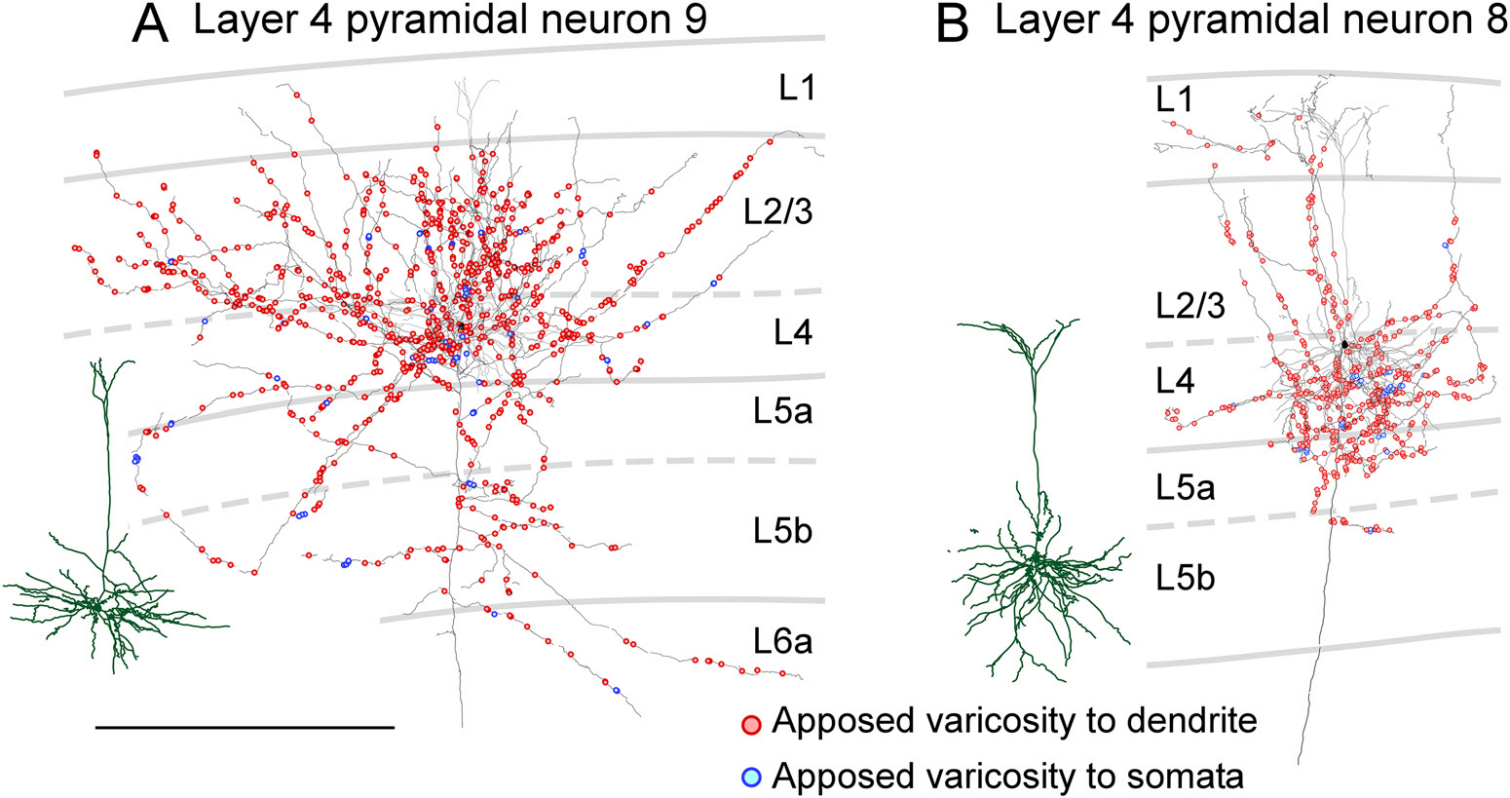
Distribution of axon varicosities of layer 4 pyramidal neurons in close appositions to PV neurons. Two representative layer 4 pyramidal neurons are shown (***A***, ***B***). Axons of pyramidal neurons were two-dimensionally reconstructed and projected onto the frontal plane. Black lines and filled circles represent axons and cell bodies of pyramidal neurons, respectively; red and blue circles indicate the axodendritic and axosomatic appositions, respectively. One each apposed varicosity is represented with a red or blue circle. Dark green and gray lines indicate reconstructed dendrites and their actual positions in the cortical layers, respectively. The other layer 4 pyramidal neurons are shown in Extended Data Figure 4-1. Scale bar: 500 μm.

In layer 4, all five reconstructed neurons were classical pyramidal neurons in the area M1 (neurons 6–8) and in the area FL/HL (neurons 9 and 10; Fig. 4; Extended Data Fig. 4-1). The largest average number of all the labeled axon varicosities per pyramidal neuron was observed for layer 4 pyramidal neurons (Table 1). The majority of axon varicosities of layer 4 pyramidal neurons were located in layers 2–4. Of the five layer 4 pyramidal neurons, three neurons (neurons 7, 9, and 10) distributed their axon varicosities plentifully in layers 2–4, while two neurons (neurons 6 and 8) distributed their axon varicosities abundantly in layer 4 but sparsely in layer 2/3 (Fig. 4; Extended Data Fig. 4-1). The percentage of apposed varicosities to all the labeled varicosities of layer 4 pyramidal neurons was 11.9–19.0% (15.3 ± 3.4%, mean ± SD).

10.1523/ENEURO.0567-20.2021.f4-1 Extended Data Figure 4-1 Distribution of axon varicosities of layer 4 pyramidal neurons in close appositions to PV neurons. Axons of layer 4 pyramidal neurons were reconstructed two-dimensionally and projected onto the frontal plane. Black lines and filled circles represent axons and cell bodies of pyramidal neurons, respectively; red and blue circles indicate the axodendritic and axosomatic appositions, respectively. Each apposed varicosity is represented by a red or blue circle. Dark green and gray lines indicate reconstructed dendrites and their actual positions in the cortical layers, respectively. Scale bar: 500 μm. Download Figure 4-1, TIF file.

**Figure 5.**
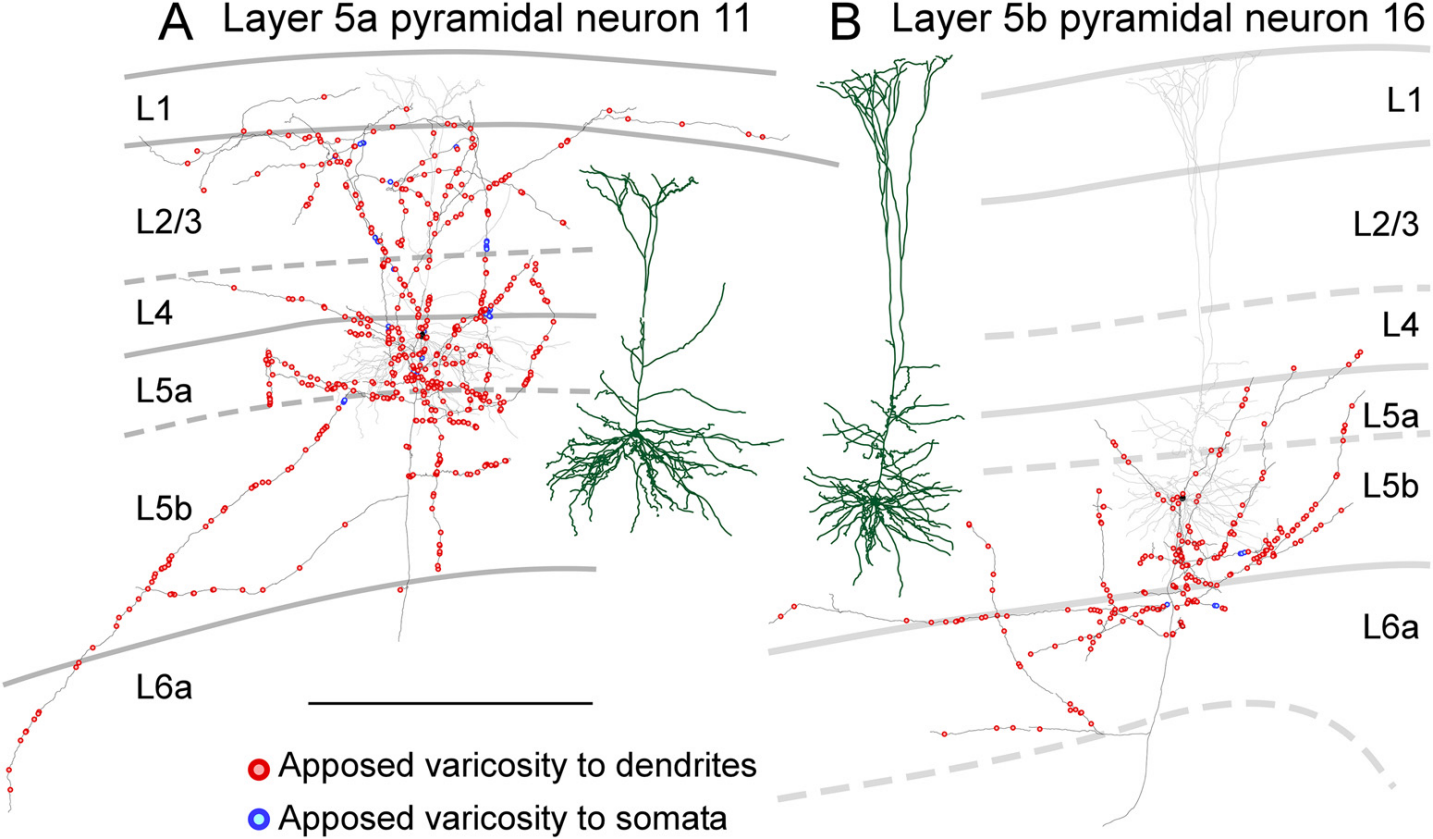
Distribution of axon varicosities of layer 5 pyramidal neurons in close appositions to PV neurons. Representative layer 5a (***A***) and layer 5b (***B***) pyramidal neurons are shown. Axons of pyramidal neurons were two-dimensionally reconstructed and projected onto the frontal plane. Black lines and filled circles represent axons and cell bodies of pyramidal neurons, respectively; red and blue circles indicate the axodendritic and axosomatic appositions, respectively. One each apposed varicosity is represented with a red or blue circle. Dark green and gray lines indicate reconstructed dendrites and their actual positions in the cortical layers, respectively. The layer 5b pyramidal neuron (***B***) had well developed apical dendrites than the layer 5a neuron (***A***). The other layer 5a and 5b pyramidal neurons are shown in Extended Data Figures 5-1, 5-2, respectively. Scale bar: 500 μm.

Layer 5a pyramidal neurons are known to have slender-tufted apical dendrites, whereas layer 5b pyramidal neurons are known to possess thick-tufted apical dendrites (Radnikow and Feldmeyer, 2018). In the present study, the mean horizontal span of apical dendrites of layer 5a pyramidal neurons was smaller than that of layer 5b (Table 1; Fig. 5; Extended Data Figs. 5-1, 5-2), but not significantly different. Layer 5a pyramidal neurons possessed widely distributed axon collaterals across layers 1–5 (Fig. 5*A*; Extended Data Fig. 5-1), whereas layer 5b pyramidal neurons had axon collaterals that were almost restricted in layers 5–6 (Fig. 5*B*; Extended Data Fig. 5-2). Consistently, the ratio of the number of axon varicosities in layers 1–4 to those in layers 5–6 was 0.92–1.52 (1.17 ± 0.31, mean ± SD) for the layer 5a pyramidal neurons and 0.00–0.26 (0.09 ± 0.15) for layer 5b pyramidal neurons (*p *=* *0.00562, two-tailed *t* test). This difference in axon distribution might lead to a systematic difference in the connection rates to PV neurons. However, the difference in the mean percentage of apposed varicosities to all the labeled varicosities between layer 5a and 5b pyramidal neurons was insignificant (12.6 ± 1.25% vs 16.6 ± 3.33%, *p *=* *0.124, two-tailed *t* test).

10.1523/ENEURO.0567-20.2021.f5-1 Extended Data Figure 5-1 Distribution of varicosities of layer 5a pyramidal neurons in close appositions to PV neurons. Axons of layer 5a pyramidal neurons were reconstructed two-dimensionally and projected onto the frontal plane. Black lines and filled circles represent the axons and cell bodies of pyramidal neurons, respectively; red and blue circles indicate the axodendritic and axosomatic appositions, respectively. Each apposed varicosity is represented by a red or blue circle. Dark green and gray lines indicate reconstructed dendrites and their actual positions in the cortical layers, respectively. These reconstructed layer 5a pyramidal neurons had less developed apical dendrites than layer 5b neurons. Scale bar: 500 μm. Download Figure 5-1, TIF file.

10.1523/ENEURO.0567-20.2021.f5-2 Extended Data Figure 5-2 Distribution of varicosities of layer 5b pyramidal neurons in close appositions to PV neurons. Axons of layer 5b pyramidal neurons were reconstructed two-dimensionally and projected onto the frontal plane. Black lines and filled circles represent axons and cell bodies of pyramidal neurons, respectively; red and blue circles indicate the axodendritic and axosomatic appositions, respectively. Each apposed varicosity is represented by a red or blue circle. Dark green and gray lines indicate reconstructed dendrites and their actual positions in the cortical layers, respectively. These reconstructed layer 5b pyramidal neurons had more abundant apical dendrites than layer 5a neurons. Scale bar: 500 μm. Download Figure 5-2, TIF file.

**Figure 6.**
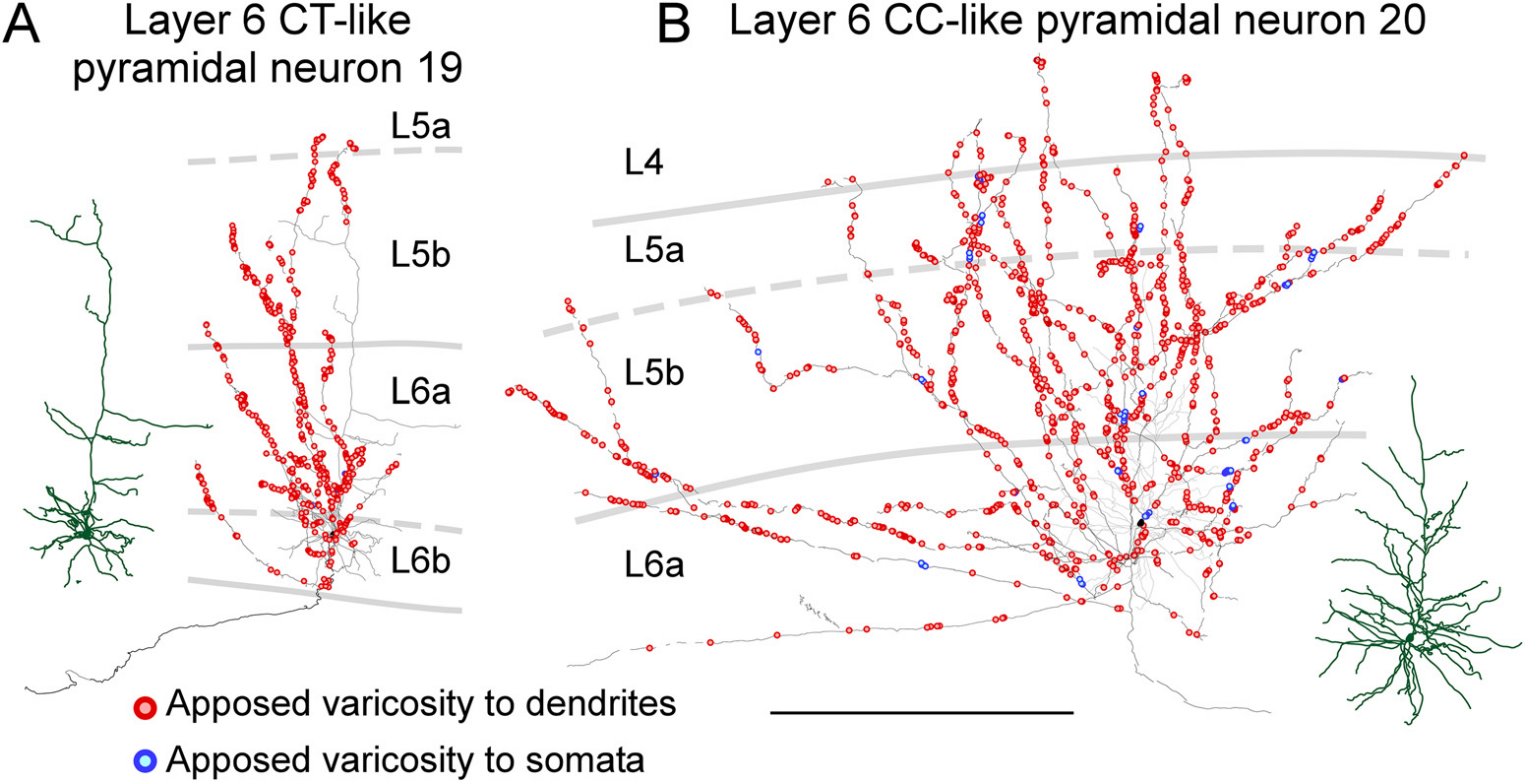
Distribution of axon varicosities of layer 6 pyramidal neurons in close appositions to PV neurons. Representative layer 6 CT-like (***A***) and CC-like (***B***) pyramidal neurons are shown. Axons of pyramidal neurons were two-dimensionally reconstructed and projected to the frontal plane. Black lines and filled circles represent axons and cell bodies of pyramidal neurons, respectively; red and blue circles indicate the axodendritic and axosomatic appositions, respectively. One each apposed varicosity is represented with a red or blue circle. Dark green and gray lines indicate reconstructed dendrites and their actual positions in the cortical layers, respectively. The other layer 6 CT-like and CC-like pyramidal neurons are shown in Extended Data Figures 6-1, 6-2, respectively. Scale bar: 500 μm.

All 10 layer 6 pyramidal neurons had short apical dendrites that ended at layer 4 and seemed to be divided into two groups based on the horizontal span of their axon collateral arbors. Five pyramidal neurons in layer 6 sent their axon collaterals vertically within 500 μm in the horizontal span, whereas the other five neurons spread their axon collaterals horizontally >500 μm (Fig. 6; Extended Data Figs. 6-1, 6-2). Three major classes of layer 6 pyramidal neurons are known to be distinguished: CT, corticocortical (CC), and cortico-claustrum neurons (Thomson, 2010; Yang et al., 2021). As defined, layer 6 CT neurons project their main axons to the thalamus and layer 6 CC neurons often have main axons projecting to other cortical areas (such as somatosensory or other motor areas). Layer 6 CT neurons are upright pyramidal neurons with narrow local axon arbors that project up toward more superficial layers, while layer 6 CC neurons include a range of atypical dendritic morphologies such as inverted pyramidal neurons, bipolar neurons, as well as upright pyramidal neurons. Unlike layer 6 CT neurons, CC neurons have long, horizontally oriented axon collaterals in widespread (Kumar and Ohana, 2008; Tanaka et al., 2011b). A third group of layer 6 pyramidal neurons project to the claustrum, and differ from both CT and CC neurons in their dendrites. Unlike other layer 6 neurons, cortico-claustrum neurons have long, slender apical dendrites that reach layer 1 (Thomson, 2010; Yang et al., 2021). In the present study, all 10 reconstructed layer 6 pyramidal neurons had short apical dendrites that ended at layer 4 or layer 5 (Fig. 6; Extended Data Figs. 6-1, 6-2), indicating that they were not cortico-claustrum neurons, but CC or CT neurons. According to the criteria by Thomson (2010), five pyramidal neurons with rich vertical axon collaterals were classified as layer 6 CT-like pyramidal neurons (Table 1; Fig. 6, left; Extended Data Fig. 6-1), whereas the other five neurons with abundant horizontal axon collaterals were classified as layer 6 CC-like pyramidal neurons (Table 1; Fig. 6, right; Extended Data Fig. 6-2).

10.1523/ENEURO.0567-20.2021.f6-1 Extended Data Figure 6-1 Distribution of varicosities of layer 6 CT-like pyramidal neurons in close appositions to PV neurons. Axons of layer 6 CT-like pyramidal neurons were reconstructed two-dimensionally and projected onto the frontal plane. Black lines and filled circles represent axons and cell bodies of pyramidal neurons, respectively; red and blue circles indicate the axodendritic and axosomatic appositions, respectively. Each apposed varicosity is represented by a red or blue circle. Dark green and gray lines indicate reconstructed dendrites and their actual positions in the cortical layers, respectively. Neurons 17 and 25 had apical dendrites that terminated in layer 4, and neurons 21 and 22 possessed apical dendrites that terminated in layer 5, suggesting that they were appeared to be Type II and Type I CT-like neurons, respectively. See the text for further details. Scale bar: 500 μm. Download Figure 6-1, TIF file.

10.1523/ENEURO.0567-20.2021.f6-2 Extended Data Figure 6-2 Distribution of varicosities of layer 6 CC-like pyramidal neurons in close appositions to PV neurons. Axons of layer 6 CC-like pyramidal neurons were reconstructed two-dimensionally and projected onto the frontal plane. Black lines and filled circles represent axons and cell bodies of pyramidal neurons, respectively; red and blue circles indicate the axodendritic and axosomatic appositions, respectively. Each apposed varicosity is represented by a red or blue circle. Dark green and gray lines indicate reconstructed dendrites and their actual positions in the cortical layers, respectively. Scale bar: 500 μm. Download Figure 6-2, TIF file.

**Figure 7.**
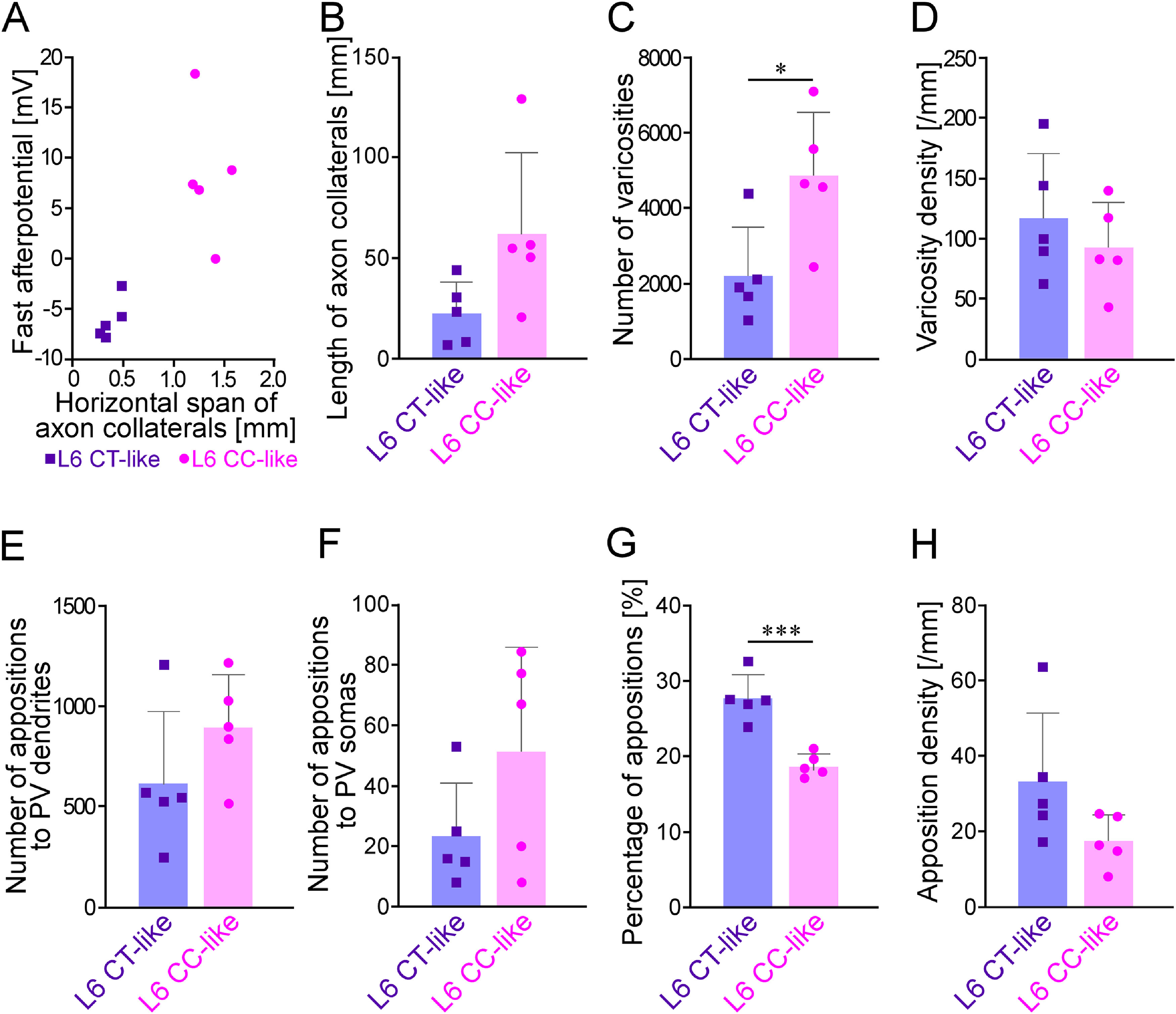
Comparisons of morphological and physiological properties between layer 6 CT-like and CC-like pyramidal neurons. ***A***, A scattergram showing the relationship between horizontal spans of axon collaterals of layer 6 pyramidal neurons and fast afterhyperpotentials. Layer 6 pyramidal neurons were subdivided into two groups based on the horizontal spans of their axon collaterals; layer 6 CT-like pyramidal neurons, which had axon collaterals restricted within 500-μm width, and layer 6 CT-like pyramidal neurons, which possessed wider axon collaterals extended >1200-μm width. All the five layer 6 CT-like pyramidal neurons showed deeper fast hyperpolarizing afterpotentials than those of the layer 6 CC-like pyramidal neurons. ***B–H***, Comparison of morphological properties between layer 6 CT-like and CC-like pyramidal neurons. The number of all the labeled varicosities of layer 6 CT-like neurons was smaller than that of layer 6 CC-like neurons (***C***). In contrast, the percentage of apposed varicosities to all the labeled varicosities of layer 6 CT-like pyramidal neurons was significantly larger than that of layer 6 CC-like pyramidal neurons (***G***). Marks, bars, and error bars in ***B–H*** indicate individual values, means, and SDs, respectively.

These two types of layer 6 neurons showed different electrophysiological properties (Table 2). In particular, the fast hyperpolarizing afterpotentials of layer 6 CT-like neurons were relatively deep and significantly different from those of the layer 6 CC-like neurons (Table 2; Fig. 7*A*), which was consistent with previous studies in which layer 6 CT and CC neurons were identified by retrograde tracers (Kaneko et al., 1995; Tanaka et al., 2011b). The morphological properties of layer 6 CT-like and CC-like neurons were compared (Fig. 7*B–H*). The number of all the labeled varicosities of layer 6 CT-like neurons was smaller than that of layer 6 CC-like neurons (Fig. 7*C*). In contrast, the percentage of apposed varicosities to all the labeled varicosities of layer 6 CT-like pyramidal neurons was 28.8 ± 3.2%, which was significantly larger than that (19.8 ± 1.5%) of layer 6 CC-like pyramidal neurons (*p *=* *0.000488, two-tailed *t* test; Fig. 7*G*). Although CT-like pyramidal neurons tended to show shorter axon collaterals than CC-like neurons, the difference was not significant (Fig. 7*B*). Furthermore, there were no significant differences in the number of appositions (Fig. 7*E*,*F*), varicosity density (Fig. 7*D*), and apposition density (Fig. 7*H*) between CT-like and CC-like pyramidal neurons. Collectively, the largest average number of apposed varicosities per pyramidal neuron was observed for layer 6 CC-like pyramidal neurons, followed by layer 4, layer 2/3, layer 6 CT-like, layer 5a, and layer 5b pyramidal neurons (Table 1), indicating that layer 6 CC-like neurons had the largest mass of input to PV neurons per single pyramidal neuron.

**Table 1:** Morphological properties of sampled pyramidal neurons

	Pyramidal neurons					
	Layer 2/3	Layer 4	Layer 5a	Layer 5b	Layer 6 CTL	Layer 6 CCL
Soma area (μm^2^)	89.7 ± 13.8	89.6 ± 14.2	121.0 ± 18.2^*†^	115.6 ± 12.8	72.8 ± 5.68^‡‡‡§§^	87.3 ± 12.7^‡^
Horizontal span of apical dendrite (μm)^*a*^	247.3 ± 13.8	162.9 ± 85.6	233.0 ± 16.1	359.2 ± 181.8	144.9 ± 38.6^§^	195.2 ± 82.0
Height of apical dendrites (μm)^*a*^	321.5 ± 70.4	414.3 ± 27.3	512.4 ± 39.3	746.5 ± 111.4^‡‡^	588.4 ± 37.8^†^	411.5 ± 83.9^§§§§¶¶^
Horizontal span of basal dendrite (μm)	306.9 ± 54.0	324.8 ± 65.8	390.7 ± 53.0	375.0 ± 90.5	273.2 ± 77.2	332.6 ± 50.1
Horizontal span of axon collaterals (mm)^*a*^	0.995 ± 0.188	1.098 ± 0.313	1.447 ± 0.409	1.245 ± 0.171	0.387 ± 0.105**^††‡‡‡‡§§§^	1.335 ± 0.164^¶¶¶¶^
Length of axon collaterals (mm)^*b*^	70.9 ± 33.8	64.6 ± 19.9	64.2 ± 15.0	16.8 ± 7.3	22.8 ± 15.5	62.5 ± 40.0
Varicosity size						
Short diameter (μm)	0.48 ± 0.14	0.46 ± 0.16	0.50 ± 0.18	0.48 ± 0.20	0.49 ± 0.15	0.45 ± 0.15
Long diameter (μm)	0.64 ± 0.20	0.64 ± 0.25	0.68 ± 0.24	0.67 ± 0.27	0.62 ± 0.17	0.63 ± 0.22
Area (μm^2^)	0.24 ± 0.13	0.24 ± 0.17	0.27 ± 0.20	0.26 ± 0.21	0.23 ± 0.13	0.24 ± 0.18
Perimeter (μm)	1.77 ± 0.53	1.74 ± 0.63	1.87 ± 0.63	1.80 ± 0.71	1.75 ± 0.48	1.72 ± 0.55
Number of varicosities (A)	5778 ± 968	5863 ± 1646	5549 ± 1289	2418 ± 1145*^†^	2227 ± 1284**^††^	5142 ± 1957
Varicosity density (/mm)	89.3 ± 24.0	94.5 ± 25.4	104.1 ± 47.3	148.8 ± 40.2	118.3 ± 28.9	93.2 ± 37.1
Number of apposed varicosities to						
PV dendrites (B)	680 ± 162	830 ± 219	635 ± 145	365 ± 121	619 ± 354	898 ± 258
PV cell bodies (C)	42 ± 16	50 ± 24	39 ± 21	19 ± 13	23 ± 18	51 ± 35
B/A (%)	11.7 ± 1.6	14.4 ± 3.4	11.9 ± 1.0	15.9 ± 3.4	27.7 ± 3.1****^††††‡‡‡‡§§§§^	18.8 ± 1.5**^‡¶¶¶^
(B+C)/A (%)	12.4 ± 1.6	15.3 ± 3.4	12.6 ± 1.3	16.6 ± 3.3	28.8 ± 3.2****^††††‡‡‡‡§§§§^	19.8 ± 1.5**^‡¶¶¶^
Apposed varicosities density (/mm)	10.2 ± 1.6	13.9 ± 5.5	10.4 ± 3.8	22.7 ± 3.7	33.4 ± 18.0**^†‡^	17.5 ± 6.9
Number of varicosities in compound appositions (D)	224 ± 89	284 ± 54	174 ± 114	120 ± 43	315 ± 205	462 ± 155^*‡§§^
D/A (%)	3.8 ± 1.1	5.0 ± 1.0	3.2 ± 1.2	5.1 ± 0.6	14.8 ± 0.2****^†††‡‡‡‡§§§^	9.7 ± 1.6^*‡^
D/B (%)	32.5 ± 7.2	35.0 ± 4.6	26.5 ± 8.7	33.0 ± 4.7	52.6 ± 2.8*^†‡‡§^	51.7 ± 7.3*^†‡‡^
Compound varicosity density (/mm)	6.9 ± 2.1	9.2 ± 2.0	5.9 ± 2.9	9.2 ± 3.6	30.2 ± 1.2****^††††§§§§^	16.4 ± 4.7**^†‡‡¶¶¶¶^

*,^†^,^‡^,^§^,^¶^ Significant differences (*,^†^,^‡^,^§^,^¶^ p < 0.05; **,^††^,^‡‡^,^§§^,^¶¶^ p < 0.01; ***,^†††^,^‡‡‡^,^§§§^,^¶¶¶^ p < 0.001; ****,^††††^,^‡‡‡‡^,^§§§§^,^¶¶¶¶^ p < 0.0001 by one-way-ANOVA and Tukey's multiple comparison test) from the value of layer 2/3, layer 4, layer 5a, layer 5b, or layer 6 CT-like (CTL) neurons, respectively.

^*a*^The horizontal span and height of dendrites or axon collaterals were measured in the frontal plane to which all the dendrites were projected.

^*b*^Length of axon collaterals was estimated by multiplying the length of axon collaterals projected onto the frontal plane by 4/*π*.

**Table 2:** Electrophysiological properties of sampled pyramidal neurons

	Pyramidal neurons					
	Layer 2/3	Layer 4	Layer 5a	Layer 5b	Layer 6 CTL	Layer 6 CCL
Resting membrane potential (mV)	-77.4 ± 6.2	-70.7 ± 7.0	-63.7 ± 8.3	-71.7 ± 9.5	-63.7 ± 6.6*	-72.2 ± 5.2
Membrane time constant (ms)	9.9 ± 1.4	14.3 ± 6.2	17.5 ± 0.5	10.3 ± 1.9	10.0 ± 4.1	7.4 ± 10.0
Input resistance (MΩ)	73.0 ± 16.4	121.4 ± 39.9	119.9 ± 38.9	63.4 ± 11.9	111.5 ± 28.5	81.3 ± 11.4
Action potential (AP) threshold (mV)	-44.7 ± 1.5	-47.5 ± 2.4	-46.1 ± 3.8	-52.8 ± 4.1	-50.3 ± 2.0	-47.2 ± 9.4
AP height (mm)^*a*^	111.9 ± 11.1	101.4 ± 10.3	90.7 ± 12.6*	99.5 ± 6.0	84.8 ± 3.2**	108.5 ± 7.6^¶¶^
AP half width (ms)	1.04 ± 0.32	1.20 ± 0.23	1.03 ± 0.11	0.76 ± 0.01	0.68 ± 0.12^†^	0.98 ± 0.20
Fast afterpotential (mV)^*b*^	2.7 ± 3.1	6.4 ± 5.8	-0.8 ± 3.3	1.7 ± 4.4	-6.1 ± 2.0^†^	8.3 ± 6.6^¶¶^
Slow afterpotential (mV)^*b*^	-0.8 ± 1.5	2.0 ± 3.4	-3.5 ± 1.0	-2.0 ± 2.3	-2.1 ± 2.1	0.7 ± 3.4

*,^†^,^‡^,^§,^^¶^Significant differences (*,^†^,^‡^,^§,^^¶^ p < 0.05; **,^††^,^‡‡^,^§§,^^¶¶^ p < 0.01; ***,^†††^,^‡‡‡^,^§§§,^^¶¶¶^ p < 0.001; ****,^††††^,^‡‡‡‡^,^§§§§,^^¶¶¶¶^ p < 0.0001 by one-way-ANOVA and Tukey's multiple comparison test) from the value of layer 2/3, layer 4, layer 5a, layer 5b, or layer 6 CT-like (CTL) neurons, respectively.

^*a*^AP height was measured form resting membrane potential.

^*b*^Fast and slow afterpotential was measured from baseline prior to action potential evoked by a short (<5 ms) depolarizing pulse, at 3.3 ms or 29 ms from action potential onset, respectively. Pulse response without action potential to the same depolarizing pulse was recorded in another trace and subtracted. Our previous study showed that one type of layer 6 pyramidal neurons has a peak of fast afterhyperpolarization (AHP) at 3.3 ms on average, and that another type of layer 6 pyramidal neurons lacks fast AHP and has a peak of medium-range AHP at 29 ms on average (Kaneko et al., 1995).

### Laminar distribution of apposed varicosities

**Figure 8.**
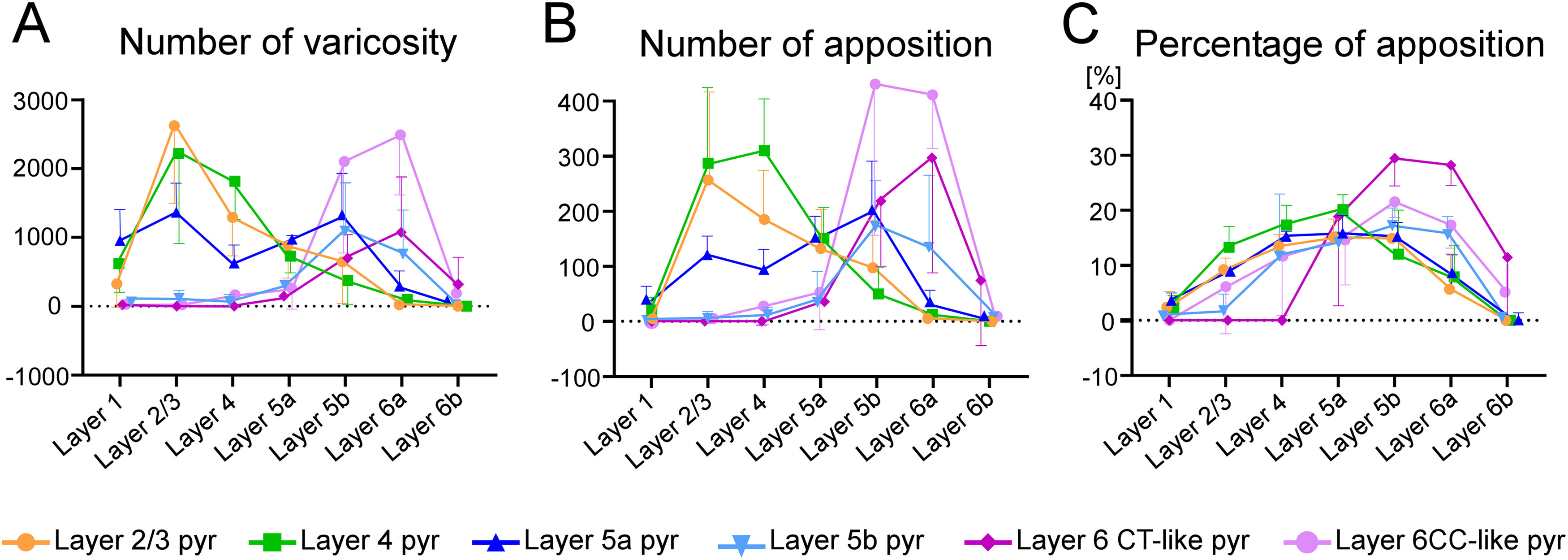
Quantitative comparisons of varicosities and appositions of pyramidal neurons in each layer. ***A***, The number of varicosities per single pyramidal neurons in each layer. Varicosities of pyramidal neurons in layers 2–5a were mainly distributed in layers 1–5a, whereas those of pyramidal neurons in layers 5b–6 were mostly located in layers 5b–6. ***B***, The number of appositions per single pyramidal neuron in each layer. In layers 1–5a, the largest average number of appositions from each pyramidal neuron to PV-neuron dendrites was observed for layer 4 pyramidal neurons, followed by layer 2/3 pyramidal neurons. Layer 6 CC-like pyramidal neurons formed the largest number of appositions to PV-neuron dendrites in layers 5b–6. ***C***, Percentage of apposed varicosities to all the labeled varicosities in each layer. Pyramidal neurons in layers 2–5a most frequently formed appositions to PV-neuron dendrites in layer 5a, whereas pyramidal neurons in layers 5b–6 most frequently apposed to PV-neuron dendrites in layers 5b and 6a. Marks and error bars in ***A–C*** indicate means and SDs, respectively.

Since axon collaterals of each pyramidal neuron showed characteristic laminar distributions (Figs. 3-6; Extended Data Figs. 3-1, 4-1, 5-1, 5-2, 6-1, 6-2), the numbers of varicosities and apposed varicosities were compared across layers (Fig. 8*A*,*B*). In this and subsequent analysis, we focused on the apposed varicosities targeting the dendrites of PV neurons because these apposed varicosities explained 94–97% of all the apposed varicosities. The distribution of the varicosities and appositions notably differed between layers 1–5a and layers 5b–6, as shown in Figure 8*A*,*B*. In layers 1–5a, the largest number of apposed varicosities per presynaptic neuron was observed for layer 4 pyramidal neurons, followed by layer 2/3, and layer 5a pyramidal neurons; in layers 5b–6, the largest number of apposed varicosities was observed for layer 6 CC-like pyramidal neurons, followed by layer 6 CT-like, layer 5b, and layer 5a pyramidal neurons. These results suggest that, on an average, single layer 2/3 and layer 4 pyramidal neurons provide a large mass of input to PV neurons in layers 1–5a, whereas individual layer 6 CC-like and CT-like pyramidal neurons provide a large amount of input to PV neurons in layers 5b–6.

The percentage of apposed varicosities to all the labeled varicosities in each layer showed characteristic patterns (Fig. 8*C*). Those of pyramidal neurons in layers 2–5a showed a curve with a peak of layer 5a, whereas those of pyramidal neurons in layers 5b–6 peaked at layers 5b and 6a. These results suggest two possibilities: (1) pyramidal neurons in layers 5b–6 form close appositions with PV neurons more frequently than other types of neurons in layers 5b and 6a, and/or (2) pyramidal neurons in layers 5b–6 formed close appositions randomly to all neuron types, however, in layers 5b and 6a, the distribution density of PV neurons was higher than that of other neurons.

**Figure 9.**
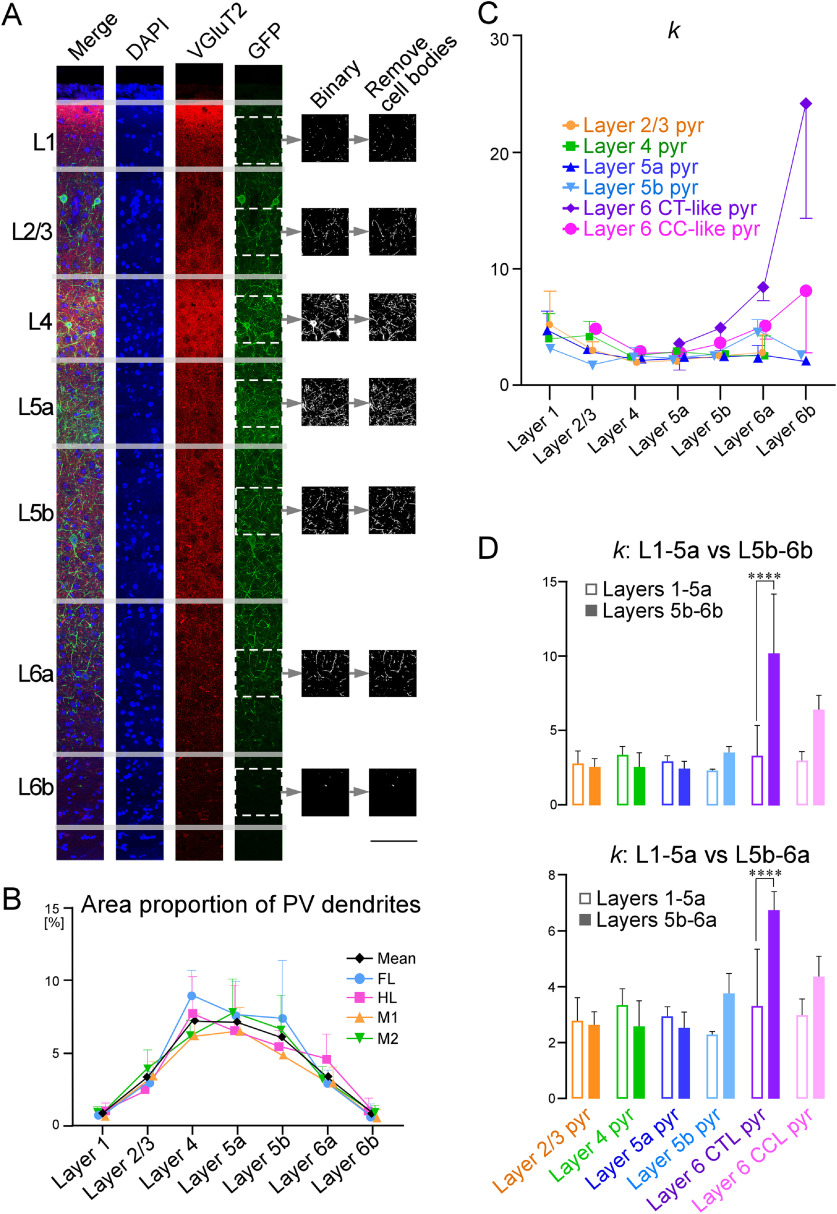
Area proportion of PV-neuron dendrites in each layer, and κ. The area proportions of PV-neuron dendrites were measured in the primary motor (M1; ***A***), secondary motor (M2), forelimb (FL), and hindlimb (HL) areas. The frontal sections from PV/myrGFP-LDLRct mice were stained with DAPI and immunostained for VGluT2 (Alexa Fluor 594) to determine cortical layer structure, and observed and captured images under a fluorescent microscope or under a confocal laser-scanning microscope (***A***). In the captured images, GFP-signal was binarized and the GFP-positive area (except cell bodies) was measured. The area proportion of PV-neuron dendrites was plotted in ***B***. Among the motor-associated areas, the area proportion of PV-neuron dendrites showed a similar distribution pattern; PV-neuron dendrites existed most densely in layer 4, followed by layer 5a and layer 5b. To quantify input maps from a pyramidal neuron to PV neurons, we introduced κ (***C***). The κ of pyramidal neurons in layers 2–5 (yellow, green, dark-blue, and peal-blue lines, respectively, in ***C***) were almost constant and around 3 in all layers, indicating that these neurons uniformly form appositions to PV-neuron dendrites in all layers. In contrast, κ for layer 6 CT-like pyramidal neurons (purple line in ***C***) was dynamically changed: κ was ∼3 in layers 1–5a, whereas κ was much larger than 3 in layers 5b–6b. The average of κ was compared between layers 1–5a and layers 5b–6b (***D***; upper graph) or layers 5b–6a (***D***; lower graph). Statistical significance was assessed with Tukey’s multiple comparison test after a two-way analysis of variance (****p <* *0.0001). Marks/bars and error bars in ***B–D*** indicate means and SDs, respectively. Scale bar: 100 μm (***A***).

If hypothesis (2) is correct, the occurrence of connections between axon varicosities and postsynaptic targets in the neuropil simply obeys the occurrence probability of both structures (Peters’ rule; Peters and Feldman, 1976; Braitenberg and Schüz, 1991; Peters and Payne, 1993). This situation was approximated using the formula, $C_{pyr-PV}^{u}\propto V_{pyr}^{u}\cdot A_{PV}^{u}$, where $C_{pyr-PV}^{u}$ is the number of closely apposed varicosities from individual pyramidal neurons to PV neurons, $V_{pyr}^{u}$ is the number of varicosities in layer *u* of each pyramidal neuron, and $A_{PV}^{u}$ is the area proportion of PV-neuron dendrites in layer *u* (for assumptions and limitations, see Materials and Methods). The constant of proportionality can be obtained from the data as $\kappa =\frac{C_{pyr-PV}^{u}}{V_{pyr}^{u}}\cdot \frac{1}{A_{PV}^{u}}$. If close appositions from a pyramidal neuron to PV neurons are formed randomly, κ should be constant in all layers and close to 1. It was observed that axon varicosities had sizes of ∼ 1 μm in diameter (Table 1) and that we counted close appositions $C_{pyr-PV}^{u}$ when any portion of the varicosities contacted the PV-neuron dendrites. In this case, the proportional constant should be slightly larger than 1, even in the random case. Nonetheless, by comparing κ, inputs from a pyramidal neuron to PV neurons can be compared among pyramidal neurons and among layers; that is, the greater the κ of a pyramidal neuron, the pyramidal neuron would prefer PV neurons. To calculate the κ, we measured the area proportion of the PV-neuron dendrites $A_{PV}^{u}$ in each layer of the motor-associated areas (Fig. 9*A*,*B*). The laminar distribution pattern of PV-neuron dendrites was similar among the motor-associated areas (M1, M2, FL, and HL). Specifically, PV-neuron dendrites were densely distributed in layers 4–5b with a peak at layer 4, followed by layer 6a and layer 2/3. Layer 1 and layer 6b contained only a few PV-neuron dendrites.

The κ of pyramidal neurons in layers 2–5 was almost constant and ∼3 across all layers (Fig. 9*C*). Thus, pyramidal neurons in layers 2–5 were considered to uniformly form close appositions with PV-neuron dendrites. The κ of layer 6 CT-like pyramidal neurons dramatically increased in layers 5b–6 with a peak in layer 6b (Fig. 9*C*; the largest κ of layer 6 CT-like pyramidal neurons = 24.4 ± 10.1, mean ± SD), suggesting that layer 6 CT-like pyramidal neurons preferentially formed close appositions with PV-neuron dendrites in layers 5b–6, but not in layers 1–5a. The difference in κ between layers 1–5a and layers 5b–6 was statistically significant for layer 6 CT-like pyramidal neurons (Fig. 9*D*, upper graph). However, since layer 6b, the lower part of layer 6, was separated from layer 6a by a cell-sparse fibrous zone (Valverde et al., 1989; Feldmeyer, 2012; Frandolig et al., 2019), it is likely that not only PV-neuron dendrites, but also the other neuronal dendrites were sparse, and that the value $A_{PV}^{6b}$, the area proportion of PV-neuron dendrites on layer 6b, might not provide a good approximation of the proportion of dendrites. Therefore, the equation, $\kappa =\frac{C_{pyr-PV}^{u}}{V_{pyr}^{u}}\cdot \frac{1}{A_{PV}^{u}}$, may not provide as an accurate estimation in layer 6b as in other layers. Thus, with κ in layer 6b excluded, we checked the differences in κ between layers 1–5a and layers 5b–6a. As a result, the differences in κ between layers 1–5a and layers 5b–6a were statistically significant for layer 6 CT-like pyramidal neurons (Fig. 9*D*, lower graph), indicating that layer 6 CT-like pyramidal neurons significantly prefer PV-neuron dendrites as postsynaptic targets in layers 5b–6.

### Compound appositions

**Figure 10.**
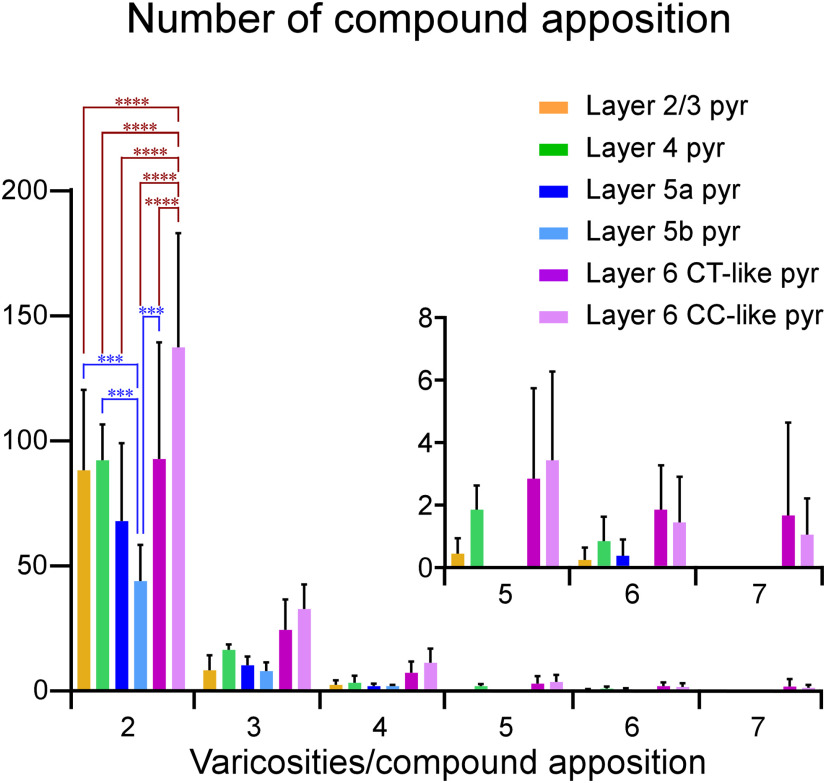
Histogram of the number of compound appositions per pyramidal neuron in each layer. Layer 6 CC-like pyramidal neurons formed the largest number of compound appositions, which consisted of two or more apposed varicosities to a dendritic branch. The inserted panel shows the amplification of the number of appositions consisting of five-seven apposed varicosities. Compound appositions consisting of seven apposed varicosities were observed only for layer 6 CT-like and CC-like pyramidal neurons. Statistical significance was assessed with Tukey’s multiple comparison test after two-way analysis of variance (***p <* *0.001, ****p <* *0.0001). Bars and error bars indicate means and SDs, respectively.

During the analysis of the closely apposed varicosities, we found that multiple appositions were located in a dendritic branch. As shown by the arrows in Figure 2*C*,*D*, five and two appositions were observed on a dendritic branch, respectively. Here, when two or more varicosities, which were derived from a single pyramidal neuron and apposed to a single dendritic branch, were observed, we tentatively named the group of appositions “compound apposition.” The number of varicosities, which participated in compound appositions, was counted, though the number may have been underestimated because the compound appositions distributed over two or more sections could not be detected by our methods. As shown in Table 1, 52–53% of the apposed varicosities of layer 6 CT-like and layer 6 CC-like pyramidal neurons contributed to compound appositions, whereas only 27–35% of those of pyramidal neurons in layers 2–5 participated in compound appositions. A single compound apposition was composed of two to seven apposed varicosities (Fig. 10). The number of compound appositions with two-apposed varicosities of layer 6 CC-like and layer 6 CT-like pyramidal neurons was significantly larger than that of the other pyramidal neurons. Compound appositions with seven apposed varicosities were only observed in layer 6 CT-like and layer 6 CC-like pyramidal neurons (Fig. 10).

## Discussion

In the mouse motor-associated cortical areas, we visualized local axon arbors of single pyramidal neurons and input sites (i.e., dendrites and cell bodies) of PV neurons using an intracellular labeling technique and transgenic mice, respectively, and examined the distribution of the local excitatory input to PV neurons based on apposed varicosities of a single pyramidal neuron to PV neurons. In layers 1–5a, the largest number of apposed varicosities per presynaptic neuron was observed for layer 4 pyramidal neurons, whereas in layers 5b–6, the largest number of apposed varicosities was found for layer 6 CC-like pyramidal neurons (Fig. 8*B*). Then, κ was introduced to quantitatively compare input maps from a pyramidal neuron to PV neurons. Layer 6 CT-like pyramidal neurons showed higher κ in layers 5b–6, suggesting preferential inputs to PV neurons (Fig. 9*C*,*D*). In contrast, other pyramidal neurons showed nearly constant κ across all layers, suggesting uniform connections to PV neurons (Fig. 9*C*,*D*). Finally, we counted the number of compound appositions, which were defined as two or more apposed varicosities to a single dendritic branch. We found that more than half of the apposed varicosities of layer 6 CT-like and CC-like pyramidal neurons contributed to the formation of compound appositions, whereas only one-third of those of pyramidal neurons in layers 2–5 participated in compound appositions (Table 1; Fig. 10). Thus, our results morphologically demonstrate that layer 6 CT-like pyramidal neurons preferentially input to PV neurons in layers 5b–6.

In the present study, we morphologically mapped local excitatory inputs from a pyramidal neuron to PV neurons and provided examples of synaptic distribution from a pyramidal neuron onto PV neurons. In contrast, previous studies have combined whole-cell clamp recording and scanning laser photo-stimulation with caged-glutamate or transfected opsins, and have examined the inputs from a group of excitatory neurons in each layer to a single FS interneuron in layer 2/3 of the somatosensory cortex (Staiger et al., 2009; Xu and Callaway, 2009), layer 4 of the somatosensory cortex (Pluta et al., 2015), layer 5 of the motor cortex (Apicella et al., 2012), and layer 6 of the visual cortex (Bortone et al., 2014). Previous results have revealed that a group of excitatory neurons in each layer most effectively drives individual PV neurons in the same or adjacent layers. The present study showed that a single pyramidal neuron in each layer mainly possessed apposed varicosities to PV neurons in the same or adjacent layers (Fig. 8*B*), supporting previous findings from a complementary viewpoint.

Recent studies have reported that layer 2/3 pyramidal neurons can be classified into layer 2 and layer 3 pyramidal neurons based on their dendritic morphology; layer 2 pyramidal neurons are characterized by a larger horizontal span in apical dendrites than in basal dendrites (van Aerde and Feldmeyer, 2015; Radnikow and Feldmeyer, 2018). Furthermore, layer 2 and layer 3 neurons have been shown to differ in terms of input, with apical dendrites of layer 2 pyramidal neurons receiving a higher density of inhibitory inputs than the apical dendrites of layer 3 pyramidal neurons (Karimi et al., 2020). Of the five layer 2/3 pyramidal neurons reconstructed in the present study, neuron 4 would be classified as a layer 2 pyramidal neuron because its apical dendrites had a larger horizontal span than its basal dendrites (Fig. 3*B*). The other four layer 2/3 neurons are considered to be layer 3 pyramidal neurons because the horizontal span of basal dendrites was larger than that of apical dendrites (Fig. 3*A*; Extended Data Fig. 3-1). Although a statistical analysis was not possible because of the small sample size, the apposition rate of the putative layer 2 pyramidal neuron to PV neurons (9.8%) was lower than that of the other presumed layer 3 pyramidal neurons (12.4–13.8%).

It has been reported that layer 5 pyramidal neurons are composed of two major groups with different output targets in the motor-associated areas. One is the pyramidal tract type, which sends axons to the brainstem and spinal cord through the pyramidal tract, and the other is the intratelencephalon type, innervating the telencephalon (Cowan and Wilson, 1994; Reiner et al., 2003; Kawaguchi, 2017). It is known that pyramidal tract type neurons, unlike intratelencephalon type neurons, have distal apical dendrites abundantly in layer 1, and that cell bodies of pyramidal tract type neurons are distributed more abundantly in layer 5b than in layer 5a (Morishima et al., 2011; Ueta et al., 2014). As the cumulative dendritic length in layer 1 of layer 5b neurons was significantly longer than that of layer 5a neurons in the present study (data not shown), the layer 5b pyramidal neurons described here were most likely pyramidal tract type neurons. However, regardless of whether the reconstructed layer 5a and layer 5b neurons were pyramidal tract type or intratelencephalon type, both pyramidal neurons in layer 5a and layer 5b formed appositions to PV neurons at similar rates, with no significant difference (Table 1). The present result is in good agreement with a previous study showing that the connection probability from pyramidal tract type neurons to FS-interneurons (0.32) was not different from the intratelencephalon type to FS-interneurons (0.22, *p *=* *0.26, Fisher’s exact test) in rats (Morishima et al., 2017).

To quantify input maps from individual pyramidal neurons to PV neurons, we introduced the parameter $\kappa =\frac{C_{pyr-PV}^{u}}{V_{pyr}^{u}}\cdot \frac{1}{A_{PV}^{u}}$. To calculate the κ, we measured the ratio of the area of PV-neuron dendrites per unit area, $A_{PV}^{u}$ in each layer of the motor-associated areas (Fig. 9*A*,*B*). If the density of total dendrites varied greatly in each layer, the $A_{PV}^{u}$ would be far from the actual ratio of the area of PV-neuron dendrites to total dendrites, and κ might not provide an accurate estimation. In particular, since layer 6b is known as a cell-sparse fibrous zone (Valverde et al., 1989; Feldmeyer, 2012; Frandolig et al., 2019), it is hypothesized that not only PV-neuron dendrites, but also the other neuron dendrites are sparse. For this reason, layer 6b was excluded from the analysis, but in layers 5b–6a, layer 6 CT-like pyramidal neurons still showed a strong preference for PV neurons ( κ = 6.6; Fig. 9*C*,*D*) than the other pyramidal neurons ( κ = 2.5–4.4; Fig. 9*C*,*D*). Even if the density of total dendrites was not uniform even outside layer 6b, the difference between layer 6 CT-like pyramidal neurons and other neurons was large, indicating the strong preference of layer 6 CT-like pyramidal neurons to PV neurons. This result is in good agreement with the previous results. It has been reported that layer 6 CT pyramidal neurons are approximately four times more likely to innervate interneurons than CC neurons and that the connection probability from CT neurons to interneuron is very high (up to 1:2) by using dual intracellular recordings in slices of the visual and somatosensory areas from adult rats and cats (Thomson et al., 2002; Mercer et al., 2005; West et al., 2006). The stronger connectivity from layer 6 CT neurons, than CC neurons, to inhibitory interneurons has been reported in the mouse motor cortex (Yamawaki and Shepherd, 2015). Furthermore, optogenetic activation of layer 6 CT neurons has been shown to enhance the activity of FS-interneurons in the mouse visual cortex (Olsen et al., 2012; Bortone et al., 2014), the auditory cortex (Guo et al., 2017), and the barrel cortex (Pauzin and Krieger, 2018; but see Frandolig et al., 2019).

Layer 6 CT-like and CC-like pyramidal neurons significantly differed in their apposition rate to PV neurons and the horizontal span to which their axon collaterals were distributed (Table 1; Fig. 7). This indicates that layer 6 CT-like pyramidal neurons might affect nearby PV neurons, whereas CC-like neurons affect a wider range of PV neurons. This is consistent with previous studies showing that optogenetic activation of CT neurons enhances the activity of FS-interneurons restricted to a narrow range (a column size) in the mouse visual cortex (Olsen et al., 2012). Layer 6 CT neurons may contribute to fine-tuning intracortical information processing within a narrow range via PV neurons.

Because of technical limitations, we could not determine the number of PV neurons innervated by a single pyramidal neuron. To reveal the number of PV neurons innervated by each pyramidal neuron, it is required to completely reconstruct the entire PV neurons. However, somas/dendrites of PV neurons located close to the surface of the slice were cut, and in many PV neurons, we could not trace and reconstruct their somas in 500-μm-thick slices. This question will be addressed by future anatomical studies using, for example, rabies virus-based retrograde monosynaptic tracing techniques (Wickersham et al., 2007; Yetman et al., 2019).

Prior studies of the rodent sensory cortex have shown the presence of two subtypes of layer 6 CT neurons with different dendritic morphology, connections and functions (Zhang and Deschênes, 1997; Zarrinpar and Callaway, 2006; Frandolig et al., 2019; Whilden et al., 2021). Type I CT neurons have apical dendrites mostly terminated in layer 5 and project their axons to both the ventroposterior medial nucleus (VPM) and posterior thalamic nucleus. In contrast, Type II CT neurons possess apical dendrites terminated in layer 4 and send their axons only to the VPM. Based on dendritic morphology, it was supposed that of five reconstructed layer 6 CT-like neurons, neurons 19, 21, and 22 with apical dendrites terminated in layer 5, and neurons 17 and 25 with apical dendrites terminated in layer 4 could be classified as Type I and Type II CT-like neurons, respectively (Fig. 6; Extended Data Fig. 6-1). Whether these CT neurons in the motor-associated areas correspond to Type I and Type II CT neurons in the sensory cortex, projecting to the different thalamic nuclei and contributing to different functions, will be addressed in future studies.

Finally, we found that more than half of the apposed varicosities of both layer 6 CT-like and CC-like pyramidal neurons contributed to the formation of compound appositions, whereas only one-third of those of pyramidal neurons in layers 2–5 participated in compound appositions (Table 1; Fig. 10). Concerning the synapses between excitatory neurons, it has been reported that “compound synapses” or “clustered synapses,” which share similar response properties and/or similar input properties and are located within a stretch of a dendritic branch of excitatory neurons, emerge in memory-related paradigms (Bloss et al., 2018; Kastellakis and Poirazi, 2019). These clustered synapses would have special significance for synaptic integration: activating a sufficient number of synapses within a single dendrite can elicit a self-regenerating, powerful dendritic spike (Losonczy and Magee, 2006). Dendritic spikes can modulate the firing of the neuron and induce localized plasticity at the dendritic level (Spruston, 2008; Hardie and Spruston, 2009; Galloni et al., 2020). In contrast, PV neurons do not initiate dendritic spikes on their dendrites and act as coincidence detectors by integrating spatially dispersed and nearly synchronous synaptic inputs (Buhl et al., 1996; Pouille and Scanziani, 2004; Glickfeld and Scanziani, 2006; Hu et al., 2010, 2014). Therefore, multiple inputs via compound appositions might be linearly integrated and contribute to rapid and reliable activation of PV neurons (Martina and Jonas, 1997; Hu et al., 2010, 2014). However, more recent studies have suggested that PV neurons can also exploit dendritic nonlinearities (Chiovini et al., 2014; Cornford et al., 2019; Tzilivaki et al., 2019). In this case, the compound appositions may perform a function different from that of linear integrators.

In conclusion, we have shown a preferential connection from layer 6 CT-like pyramidal neurons to PV neurons in the motor-associated areas based on morphologic data. PV neurons are involved in basic microcircuit functions such as feedforward and feedback inhibition (Buzsàki and Eidelberg, 1981; Miles, 1990; Pouille and Scanziani, 2001, 2004) or γ-frequency oscillations (Cardin et al., 2009; Sohal et al., 2009), and through these functions, they are also involved in more complex network operations. For example, in the sensory cortex, PV neurons have been shown to contribute to gain modulation on visual processing (Olsen et al., 2012; Bortone et al., 2014), dynamical switching between sound detection and discrimination mode of the auditory cortex (Guo et al., 2017), and refining of tactile encoding in the barrel cortex (Pauzin and Krieger, 2018). In the motor-associated areas, the connection from layer 6 CT neurons to PV neurons should also be involved in complex network operations. Our findings provide a detailed circuit basis for further elucidating the functions of the selective connection between layer 6 CT and PV neurons in the motor-associated areas.

## Synthesis

Reviewing Editor: Ayako Watabe, Jikei University School of Medicine Decisions are customarily a result of the Reviewing Editor and the peer reviewers coming together and discussing their recommendations until a consensus is reached. When revisions are invited, a fact-based synthesis statement explaining their decision and outlining what is needed to prepare a revision will be listed below. The following reviewer(s) agreed to reveal their identity: Kathleen Rockland.

We appreciate that the authors have revised the manuscript substantially according to the reviewers’ comments. However, there are insufficient responses to some of the concerns, and there are still quite a few logical leaps and points with no scientific bases, making the manuscript difficult to follow. In addition, English editing is strongly recommended. Our concerns are as follows.

Major points;

The logical structure of the Abstract, Introduction, and Discussion is not well organized, and the authors need to consider generalizations more carefully.

1, Line 45 “cortical excitatory circuits";

Because circuits are not like excitatory and inhibitory, so the authors need to delete “excitatory"

2, Lines 45-46 “and cortical excitatory circuits transfer the received information into layer 2/3 and finally into the primary output layers, layers 5 and 6";

This is an assumption and to some extent “jargon”. We recommend to change into “and intrinsic cortical circuits transmit information into layer 2/3 and thence to the primary output layers, layers 5 and 6"

3, Line 49 “that synergistically build layer-specific local circuits";

On what grounds can they claim as “synergistic”? Aren’t there translaminar circuits in addition to layer-specific ones?

4, Lines 4-7 “Since each cortical layer has a particular input-output organization and processes information in a layer-specific manner, elucidating laminar differences in connections between pyramidal and PV neurons will help understand the role of PV neurons in such layer-specific information processing.”;

This sentence should be changed to “Since each cortical layer processes information in a layer-specific manner, elucidating laminar differences in connections between pyramidal and PV neurons will help to understand the role of PV neurons in functional processing.”

5, Line 591 ;

Consider adding: “Continued studies might also address the issue of further subtypes within the CT population (see, for example, Whilden, Chevee, An, and S.P. Brown, 2021).”

6, Lines 614-619 “In conclusion, we have shown the strong connection from layer 6 CT-like pyramidal neurons to PV neurons in the motor-associated areas based on the morphological data. In the sensory cortex, layer 6 CT neurons have been shown to activate layer 6 translaminar PV neurons sending their axon fibers vertically up to layer 1 and suppress the activity of all the layers’ pyramidal neurons of the cortical column and contribute to columnar-information processing (Bortone et al., 2014; Guo et al., 2017; Pauzin and Krieger, 2018). ;

This sentence should be changed to

"In conclusion, we have shown the strong connection from layer 6 CT-like pyramidal neurons to PV neurons in the motor-associated areas based on the morphological data. In the sensory cortex, layer 6 CT neurons have been shown to activate layer 6 translaminar PV neurons sending their axon fibers vertically up to layer 1. This morphology is proposed to suppress the activity of all the layers’ pyramidal neurons within a vertical cortical column and contribute to columnar-information processing (Bortone et al., 2014; Guo et al., 2017; Pauzin and Krieger, 2018). “

## Author Response

Correction List

We would like to express our appreciation to the referees for their suggestions on how to improve our manuscript. We have carefully considered the editor’s and referees’ comments in the email dated June 9thand replied to them point-by-point. Further, we have considered the editor’s and referees’ comments in the email dated January 30th. The changes from the previous version are indicated in blue in the revised manuscript. The number of lines in this correction list indicates the number of lines in the revised manuscript.

Synthesis Statement for Author:

We appreciate that the authors have revised the manuscript substantially according to the reviewers’ comments. However, there are insufficient responses to some of the concerns, and there are still quite a few logical leaps and points with no scientific bases, making the manuscript difficult to follow. In addition, English editing is strongly recommended. Our concerns are as follows.

Major points;

The logical structure of the Abstract, Introduction, and Discussion is not well organized, and the authors need to consider generalizations more carefully.

Answer:

We agree with the comment. We have reviewed and rewritten the sections: Abstract, Significance Statement, Introduction, Results, and Discussion. Although many suggestions were made for correcting the sentences in the previous version, during the course of revision, we found that many sentences had to be replaced with new ones. Thus, we could not directly adopt your suggestions to in the revised manuscript. Nonetheless, we have tried to understand your insightful comments and reflect them in the new version of the manuscript. Lastly, we have sought the services of an editing company, Editage, and we believe the manuscript has better readability now.

The following are the four major changes we made in the revised manuscript.

1. In the comment in the email dated January 30th, it was pointed out that “The writing style of the result section is a bit problematic and not common. The first paragraph in the Result is uncommon. It is readable, but very “dense” writing, as if someone trying to fit all of their data into an abstract that is limited to 250 words. The authors should concisely describe what they did, and then provide the reasons (i.e., the hypotheses) that they examined. Also, each Figure should be explained in a paragraph, in which the reasons, results, and perhaps conclusion/discussion should be described succinctly.” Therefore, we have rewritten the Results section according to the advice.

It has been revised to explain one Figure in one paragraph. As a result, the previous Figure 3 has been changed to Extended Data Figure 1-1 in the revised manuscript. We have explained the morphology of layer 2/3, layer 4, and layer 5 pyramidal neurons in separate paragraphs, citing figures for each one (Lines 364-436). In addition, Figure 7 has been added to clarify the classification of layer 6 CT-like and CC-like neurons in the revised manuscript. Therefore, Figures 7-9 have been renumbered to Figures 8-10, respectively, in the revised manuscript. In the previous Results section, there was a paragraph entitled “Horizontal distribution of apposed varicosities”. We have moved this paragraph to the Discussion section as we considered it more appropriate for the Discussion section in the revised manuscript (lines 614-638). As described above, the text and the structure of the Results section have been modified significantly, but the data remain unchanged.

2. Regarding the Discussion, the following was pointed out in the email dated January 30th.

"Discussion should include a summary of the results paragraph, succinct discussion related to results, and future direction. The discussion section is not well organized in that sense. Additionally, the author discussed some irrelevant topic which may not be perfectly related to the present anatomical observations.”

We agree with the comment. Therefore, we have deleted the paragraph about layer 6 translaminar PV neurons from the revised manuscript since the present results do not show the presence of layer 6 translaminar PV neurons in the motor-associated areas. Instead, we have made the following discussion on compound appositions based on our morphological observations (lines 652-675):

"Finally, we found that pyramidal neurons formed “compound appositions” on the PV-neuron dendrites. In the present study, compound appositions were defined as two or more apposed varicosities derived from a single pyramidal neuron located in a row within a dendritic branch of a PV neuron. More than half of the apposed varicosities of layer 6 CT-like and CC-like pyramidal neurons contributed to the formation of compound appositions, whereas only one-third of those of pyramidal neurons in layers 2-5 participated in compound appositions (Table 1; Fig. 10). Concerning to synapses between excitatory neurons, recently it has been reported that “compound synapses” or “clustered synapses", which share similar response properties and/or similar input properties, reside on spines located in a stretch of a dendritic branch of principal excitatory neuron, emerge in memory-related paradigms (Bloss et al., 2018; Kastellakis and Poirazi, 2019). These clustered synapses would have special significance for synaptic integration: activating a sufficient number of synapses within a single dendrite can elicit a self-regenerating, powerful dendritic spike (Losonczy and Magee, 2006). Dendritic spikes can strongly modulate the firing of the neuron and can induce localized plasticity at the dendritic level (Spruston, 2008; Hardie and Spruston, 2009). In contrast, PV neurons have been known to initiate no dendritic spikes on their dendrites, either by dendritic current injection or by synaptic stimulation and act as coincidence detectors by integrating spatially dispersed and nearly synchronous synaptic inputs (Buhl et al., 1996; Glickfeld and Scanziani, 2006; Hu et al., 2010, 2014; Pouille and Scanziani, 2004). Therefore, multiple inputs from compound appositions might be linearly integrated on a PV neuron and contribute to the rapid, reliable, and temporally precise activation of the PV neuron (Hu et al., 2010, 2014; Martina and Jonas, 1997). However, more recent studies have suggested that PV neurons can also exploit dendritic nonlinearities with supralinear calcium accumulation and sodium spikes (Chiovini et al., 2014; Tzilivaki et al., 2019; Cornford et al., 2019). In this case, the compound appositions may perform a different function beyond being linear integrators.”

Further, we added a discussion on the density of apposed varicosities as follows (lines 614-638):

"As a result, the apposition rate and connection property of layer 6 CT-like pyramidal neurons to PV neurons (27.7% and κ = 6.6, respectively) were found to be approximately 1.6 times higher than those of CC-like neurons (18.8% and κ = 4.4, respectively). However, this fact alone may not be sufficient to explain why layer 6 CT-neurons exhibit a four-fold higher binding probability to interneurons compared to CC-like neurons (Thomson, 2002; Mercer et al., 2005; West et al., 2006). The high binding probability may also be due to the high distribution density of the apposed varicosities of layer 6 CT-like pyramidal neurons onto PV neurons. The distribution density of appositions per unit volume was calculated by dividing the number of appositions by the volume in which axon collaterals were distributed, and the volume can be approximated by the following equation: volume = horizontal span of axon collaterals × vertical span of axon collaterals × thickness of slices. Here, almost all the axon collaterals of layer 6 CT-like and CC-like pyramidal neurons were distributed in layers 5-6, and the height of layers 5-6 was approximately 0.8 mm (Fig. 6; Extended Data Fig. 6-1, 6-2). In the present study, the thickness of the slices was 0.5 mm. Therefore, the mean density of appositions was calculated as follows: for layer 6 CT-like neurons, 619/(0.387 × 0.8 × 0.5) = 3999/mm3; for layer 6 CC-like neurons, 898/(1.335 × 0.8 × 0.5) = 1682/mm3 (Table 1). The density of appositions was estimated to be 2.4 times (3999/1682 = 2.4) higher in layer 6 CT-like pyramidal neurons than in CC-like neurons. Considering the preponderance of both the density of apposition and the apposition rate, this could explain the high binding probability of CT-like pyramidal neurons to PV neurons. This estimation also suggests that each layer 6 CT-like pyramidal neuron would have a strong influence on PV neurons in the vicinity, while each CC-like pyramidal neuron moderately affects PV neurons in widespread. It has been reported that optogenetic activation of layer 6 CT neurons enhances the activity of FS-interneurons restricted to a narrow range (a column size) of the mouse visual cortex (Olsen et al., 2012). Layer 6 CT neurons may contribute to the fine-tuning of intracortical information processing within a narrow range via PV neurons.”

Furthermore, we added a discussion on the possibility that the dendritic density of all neurons is not uniform across the layers of the cortex and its effect on k, as follows (lines 601-613):

"To calculate the κ, we measured the ratio of the area of PV-neuron dendrites per unit area A_PV^uin each layer of the motor-associated areas (Fig. 9A, B). If the density of total dendrites varied greatly in each layer, the ratio of the area of PV-neuron dendrites per unit area, A_PV^u, would be far from the ratio of the area of PV-neuron dendrites to total dendrites, and κ might not provide an accurate estimation. In particular, since layer 6b is known as a cell-sparse fibrous zone (Valverde et al., 1989; Feldmeyer 2012; Frandolig et al. 2019), it is hypothesized that not only PV-neuron dendrites, but also the other neuron dendrites are sparse. For this reason, layer 6b was excluded from the analysis, but in layers 5b-6a, layer 6 CT-like pyramidal neurons still showed a strong preference for PV neurons (κ = 6.6; Fig. 9C, D) than the other pyramidal neurons (κ = 2.5-4.4; Fig. 9C, D). Even if the density of total dendrites was not uniform even outside layer 6b, the difference between layer 6 CT-like pyramidal neurons and other neurons was large, indicating the strong preference of layer 6 CT-like pyramidal neurons to PV neurons.”

3. We have rewritten the introduction.

This study aimed to compare the connection properties from different types of pyramidal neurons (especially layer 6 CT-like and CC-like neurons) to PV neurons. Different types of pyramidal neurons form local circuits with different input-output relationships, and inhibitory neurons also play an important role in local circuits. It is necessary to analyze the electrophysiological connection properties of pyramidal neurons and PV neurons as well as the underlying structures to understand the function of PV neurons in local circuits. Using the forementioned logic, we have rewritten the Introduction (lines 36-56).

Furthermore, we have indicated the points that have been clarified in the previous studies and those that remain unknown. Previous studies have investigated the effects of pyramidal neurons on a single PV neuron. Conversely, it remains unknown how a single pyramidal neuron innervates PV neurons. Although a previous morphological study has shown that layer 6 CT neurons are strongly connected to interneurons, there is no clarity regarding postsynaptic neuron types, except that they are interneurons.

With these points in mind, we have tried to explain the methodology of this study and what we have uncovered carefully (lines 57-93).

4. Due to the aforementioned changes, the Abstract and the Significance Statement sections have been rewritten.

The Abstract is shown in the lines 1-20 and the Significance Statement appears in the lines 26-35.

Reviewer comments:

1, Line 45 “cortical excitatory circuits";

Because circuits are not like excitatory and inhibitory, so the authors need to delete “excitatory”

Answer:

We agree with your opinion, “circuits are not like excitatory and inhibitory.” This study aimed to determine whether different types of pyramidal neurons have different connection properties to PV neurons. Therefore, the flow of information processing by layers is not relevant to this study and we have deleted the sentence pointed out. We apologize for not being able to adopt this in the revised manuscript. Instead, we have replaced it with the explanation that each type of pyramidal neuron has its own unique input/output characteristics and forms local circuits, including GABAergic neurons.

The rewritten text is as follows (lines 41-46):

"The principal excitatory neurons in each layer show cell-type-specific input-output connectivities and form local circuits that enable cortical computations (Shepherd and Yamawaki, 2021; Kaneko, 2013). The local cortical circuit contains 20-30% of inhibitory GABAergic interneurons, which are essential for the stabilization of network dynamics (Tsodyks et al., 1997) and for improving the efficiency and robustness of the neural code (Denève and Machens, 2016; Renart et al., 2010; Somers et al., 1995).”

2, Lines 45-46 “and cortical excitatory circuits transfer the received information into layer 2/3 and finally into the primary output layers, layers 5 and 6";

This is an assumption and to some extent “jargon”. We recommend to change into “and intrinsic cortical circuits transmit information into layer 2/3 and thence to the primary output layers, layers 5 and 6"

Answer:

The flow of information processing by layers is not relevant to this study, and we have deleted the sentence pointed out. We apologize for not being able to adopt this in the revised manuscript. Instead, we have replaced it with the explanation that each type of pyramidal neuron has its own unique input/output characteristics and forms local circuits, including GABAergic neurons.

The rewritten text is shown in lines 41-46.

3, Line 49 “that synergistically build layer-specific local circuits";

On what grounds can they claim as “synergistic”? Aren’t there translaminar circuits in addition to layer-specific ones?

Answer:

We apologize for the inappropriate wording, “synergistic”. There is no evidence to support “synergistic.”

As stated, there are layer-specific circuits as well as translaminar circuits. Therefore, we have rewritten the sentence pointed out as follows (lines 43-46):

"The local cortical circuit contains 20-30% of inhibitory GABAergic interneurons, which are essential for the stabilization of network dynamics (Tsodyks et al., 1997) and for improving the efficiency and robustness of the neural code (Denève and Machens, 2016; Renart et al., 2010; Somers et al., 1995).”

4, Lines 4-7 “Since each cortical layer has a particular input-output organization and processes information in a layer-specific manner, elucidating laminar differences in connections between pyramidal and PV neurons will help understand the role of PV neurons in such layer-specific information processing.”;

This sentence should be changed to “Since each cortical layer processes information in a layer-specific manner, elucidating laminar differences in connections between pyramidal and PV neurons will help to understand the role of PV neurons in functional processing.”

Answer:

Thank you for your suggestion. The purpose of this study was to clarify the binding properties of each type of pyramidal neuron to PV neurons and speculate on the function of these PV neurons in local circuits. Therefore, we have rewritten the sentence pointed out as follows (lines 54-56). We apologize for not being able to adopt the sentence stated above.

"Given that the function of a neural circuit is restricted by its particular connectivity, clarifying the specific connectivity rules between pyramidal and PV neurons is essential for understanding the role of PV neurons in local circuits.”

5, Line 591; Consider adding: “Continued studies might also address the issue of further subtypes within the CT population (see, for example, Whilden, Chevee, An, and S.P. Brown, 2021).”

Answer:

Thank you for your suggestion.

We have cited the paper shown as follows (lines 647-651):

"Recently, the presence of further subtypes within the CT neuronal population in the sensory cortex has been reported, and these subtypes are likely to have different connection properties and functions (Whilden et al., 2021; Frandolig et al., 2019). Whether these subtypes of CT neurons also exist in the motor-associated areas, and if so, how they form connections and what functions they perform, continued studies might also address these issues.”

6, Lines 614-619 “In conclusion, we have shown the strong connection from layer 6 CT-like pyramidal neurons to PV neurons in the motor-associated areas based on the morphological data. In the sensory cortex, layer 6 CT neurons have been shown to activate layer 6 translaminar PV neurons sending their axon fibers vertically up to layer 1 and suppress the activity of all the layers’ pyramidal neurons of the cortical column and contribute to columnar-information processing (Bortone et al., 2014; Guo et al., 2017; Pauzin and Krieger, 2018).

This sentence should be changed to

"In conclusion, we have shown the strong connection from layer 6 CT-like pyramidal neurons to PV neurons in the motor-associated areas based on the morphological data. In the sensory cortex, layer 6 CT neurons have been shown to activate layer 6 translaminar PV neurons sending their axon fibers vertically up to layer 1. This morphology is proposed to suppress the activity of all the layers’ pyramidal neurons within a vertical cortical column and contribute to columnar-information processing (Bortone et al., 2014; Guo et al., 2017; Pauzin and Krieger, 2018). “

Answer:

Thank you for your suggestion. The presence of layer 6 translaminar PV neurons in the motor-associated areas remains unclear in this study. Thus, we have deleted the sentence pointed out, as we consider that the discussion should be based on the present results. We apologize for not being able to adopt the proposed correction. The concluding paragraph has been rewritten as follows (lines 676-689):

"In conclusion, we have shown a strong connection from layer 6 CT-like pyramidal neurons to PV neurons in the motor-associated areas based on morphological data. PV neurons are involved in basic microcircuit functions, such as feedforward and feedback inhibition (Busáki and Eidelberg, 1981; Miles, 1990; Pouille and Scanziani, 2001, 2004) or gamma-frequency oscillations (Cardin et al., 2009; Sohal et al., 2009), and through these microcircuit functions, they are also involved in more complex network operations. In the sensory cortex, the strong connection from layer 6 CT-like pyramidal neurons to PV neurons has been shown to contribute to various complex network operations, such as gain modulation on visual processing (Olsen et al., 2012; Bortone et al., 2014), dynamical switching between sound detection mode and discrimination mode of the auditory cortex (Guo et al., 2017), and refining of tactile encoding in the barrel cortex (Pauzin and Krieger, 2018). The strong circuitry from layer 6 CT-like pyramidal neurons to PV neurons must also be performing complex network operations in the motor-associated areas. Our findings provide a detailed circuit basis for further elucidating the functions of the selective connection between layer 6 CT and PV neurons in the motor-associated areas.”
