# Supplementary figures and images for: Local Connections of Pyramidal Neurons to Parvalbumin-Producing Interneurons in Motor-Associated Cortical Areas of Mice
Source: eNeuro. 2022 Feb 2;9(1):ENEURO.0567-20.2021. doi: 10.1523/ENEURO.0567-20.2021 (PMC8856719; doi:10.1523/ENEURO.0567-20.2021)

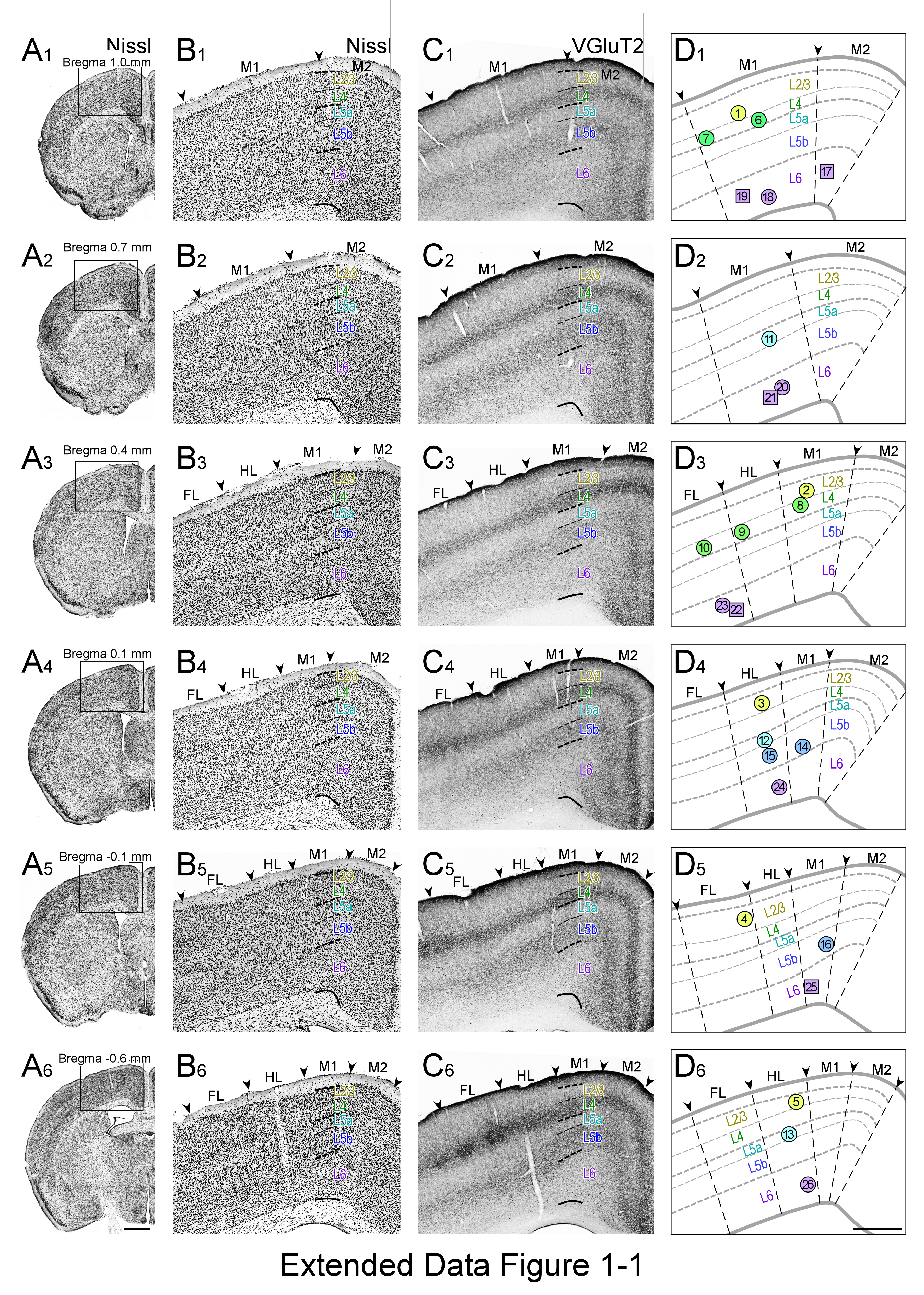

Supplement: Extended Data Figure 1-1 — The somal locations of intracellularly labeled pyramidal neurons in the present study. The somal locations were projected onto the nearest frontal plane of Nissl-stained and VGlu-stained sections and serially numbered from the superficial layer to the deep layer, from the rostral to caudal, and from the medial to lateral portions of the motor-associated cortical areas. The horizontal broken lines indicate the border of cortical layers, and the vertical broken lines indicate the border of cortical areas, which were determined in the Nissl-stained sections with the aid of VGlu immunoreactivity in the adjacent sections. Yellow, green, peal-blue, blue, and purple marks indicate somal locations of layer (L)2/3, L4, L5a, L5b, and L6 pyramidal neurons, respectively. The purple-filled circles and rectangles indicate layer 6 CC-like and CT-like pyramidal neurons, respectively. FL, forelimb region of the primary somatosensory motor area; HL, hindlimb region of the primary somatosensory motor area; M1, the primary motor area; M2, the secondary motor area. Scale bars: 1 mm (in A6; applies to A1–A6) and 500 μm (in D6; applies to B1–D6). Download Figure 1-1, TIF file. [file enu-eN-NWR-0567-20-s02.tif]

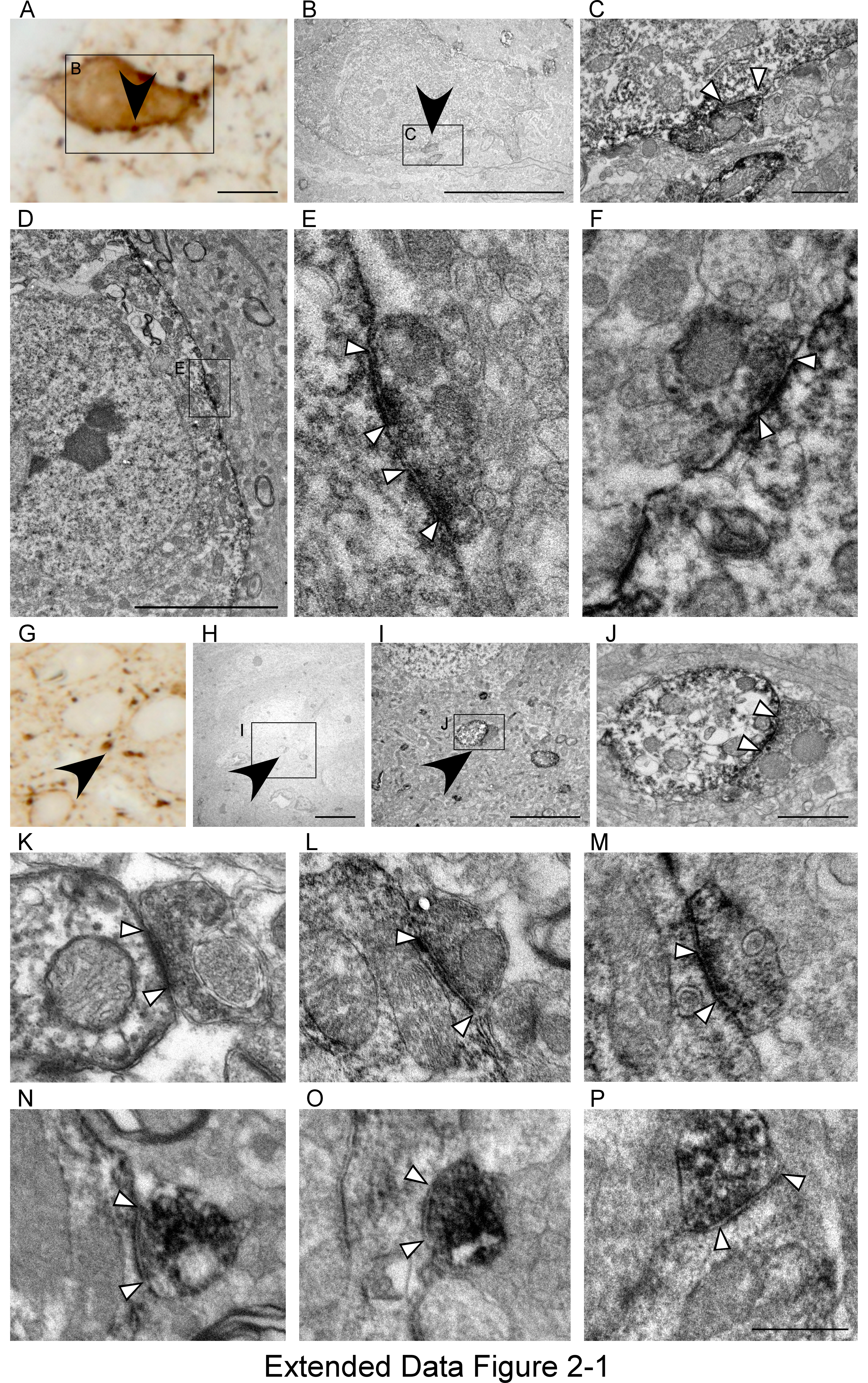

Supplement: Extended Data Figure 2-1 — Close appositions found under a light microscope were examined using electron microscopy. GFP and biocytin were visualized as brown and blue-black with the DAB and DAB/nickel reactions, respectively (A, G). Black arrowheads in A, B, G–I indicate close appositions. Axosomatic (A, B) and axodendritic (G–I) appositions were found to form asymmetrical synapses (C, J, respectively). White arrowheads in C, E, F, J–P indicate the typical asymmetric synaptic contacts that were made between the biocytin-labeled axon terminals (AT) and the cell bodies (CB) or the dendrites (Den) with GFP immunoreactivity. Scale bars: 10 μm (A), 10 μm (B), 1 μm (C), 5 μm (D), 10 μm (in H; applies to G, H), 5 μm (I), 1 μm (J), and 500 nm (in P; applies to E, F, K–P). Download Figure 2-1, TIF file. [file enu-eN-NWR-0567-20-s03.tif]

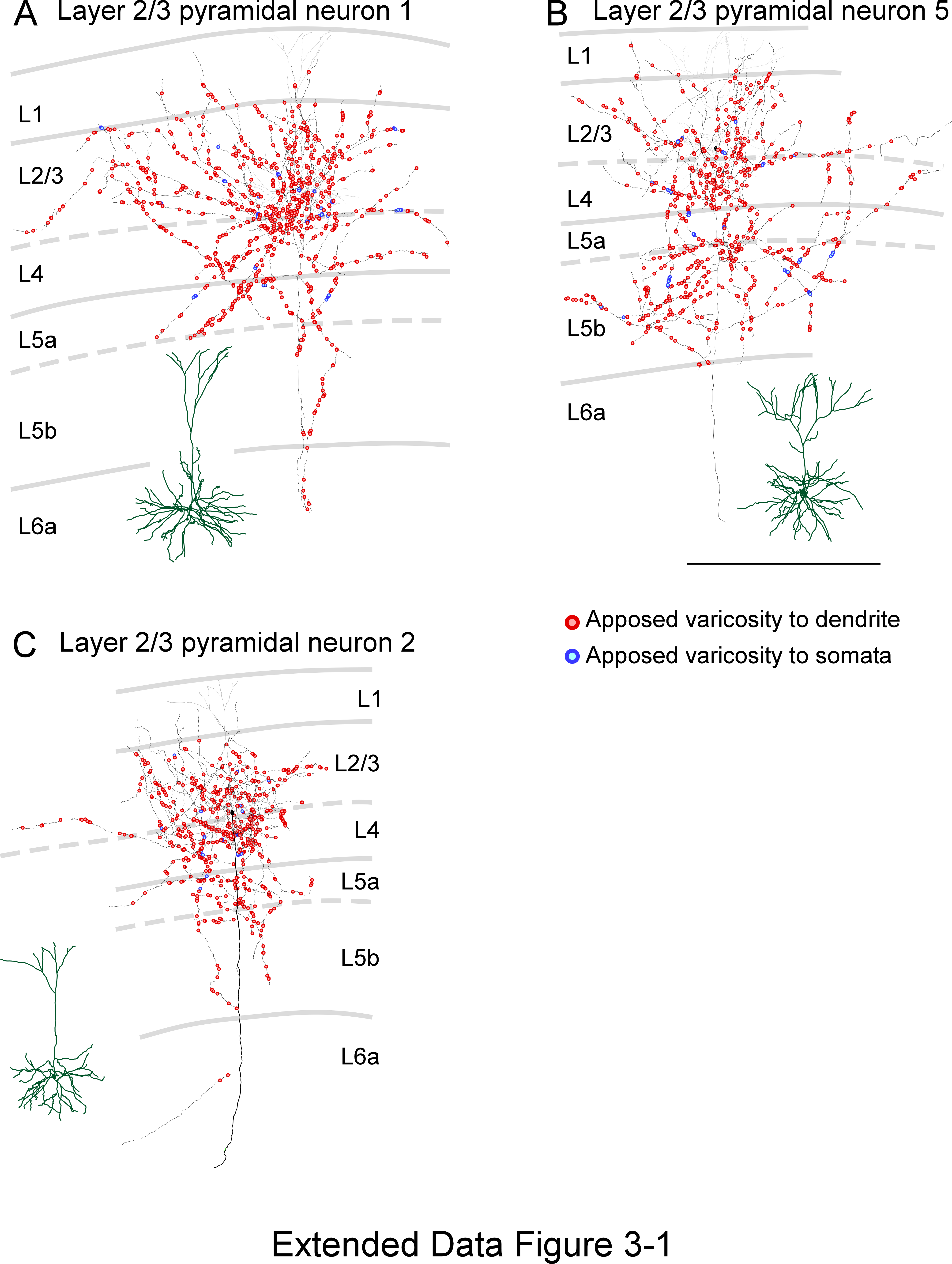

Supplement: Extended Data Figure 3-1 — Distribution of axon varicosities of layer 2/3 pyramidal neurons in close appositions to PV neurons. Axons of layer 2/3 pyramidal neurons were reconstructed two-dimensionally and projected onto the frontal plane. Black lines and filled circles represent axons and cell bodies of pyramidal neurons, respectively; red and blue circles indicate the axodendritic and axosomatic appositions, respectively. Each apposed varicosity is represented by a red or blue circle. Dark green and gray lines indicate reconstructed dendrites and their actual positions in the cortical layers, respectively. Recently, layer 2/3 pyramidal neurons are suggested to be divided into layer 2 and layer 3 neurons based on the morphology of their dendrites. In layer 2 neurons, the horizontal span of the apical dendrites is larger than that of the basal dendrites, whereas in layer 3 neurons, the horizontal span of the basal dendrites is larger. According to this criterion, the layer 2/3 neurons shown here are presumed to be layer 3 neurons because the horizontal span of their basal dendrites is larger than that of apical dendrites. Scale bar: 500 μm. Download Figure 3-1, TIF file. [file enu-eN-NWR-0567-20-s04.tif]

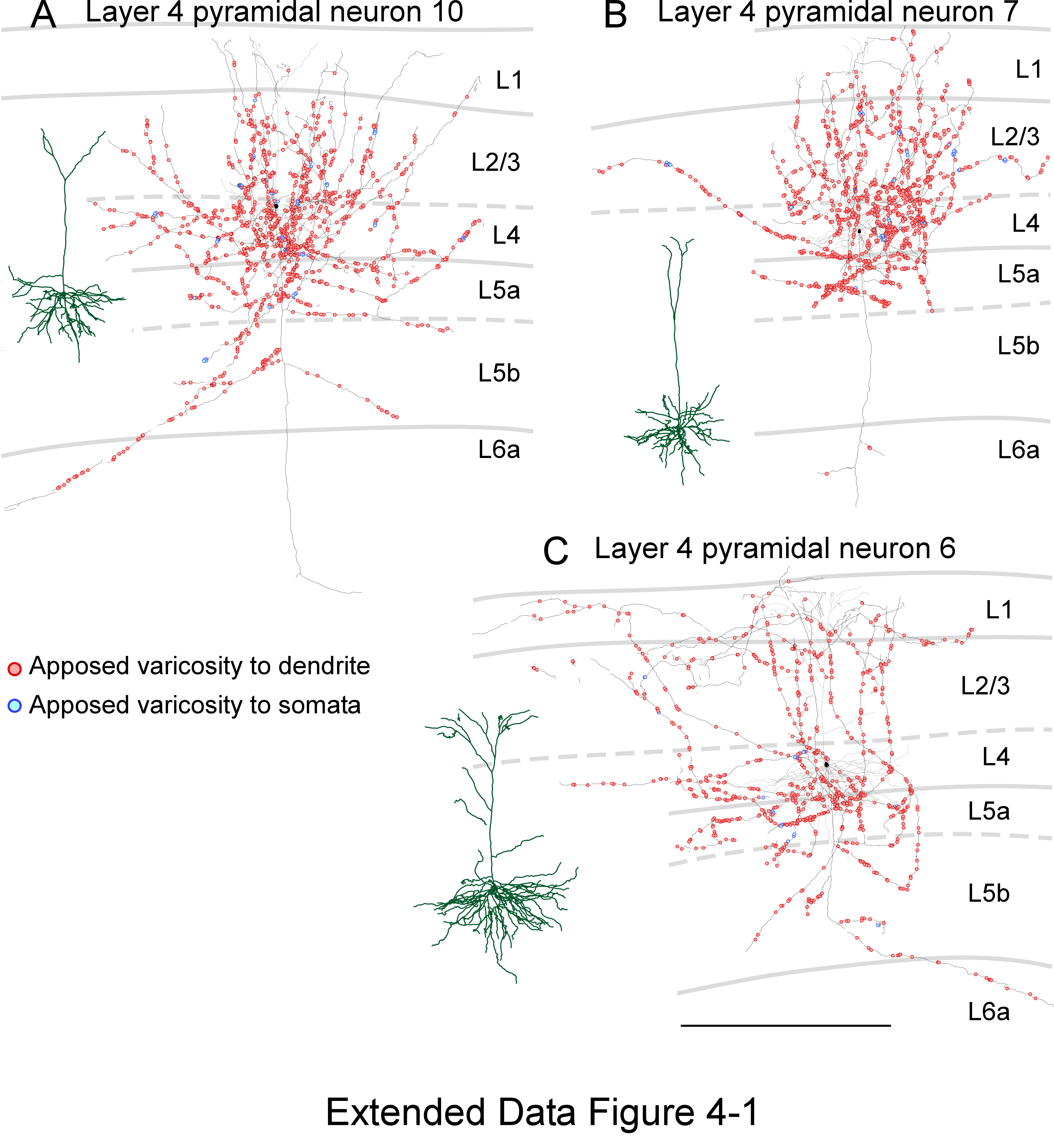

Supplement: Extended Data Figure 4-1 — Distribution of axon varicosities of layer 4 pyramidal neurons in close appositions to PV neurons. Axons of layer 4 pyramidal neurons were reconstructed two-dimensionally and projected onto the frontal plane. Black lines and filled circles represent axons and cell bodies of pyramidal neurons, respectively; red and blue circles indicate the axodendritic and axosomatic appositions, respectively. Each apposed varicosity is represented by a red or blue circle. Dark green and gray lines indicate reconstructed dendrites and their actual positions in the cortical layers, respectively. Scale bar: 500 μm. Download Figure 4-1, TIF file. [file enu-eN-NWR-0567-20-s05.tif]

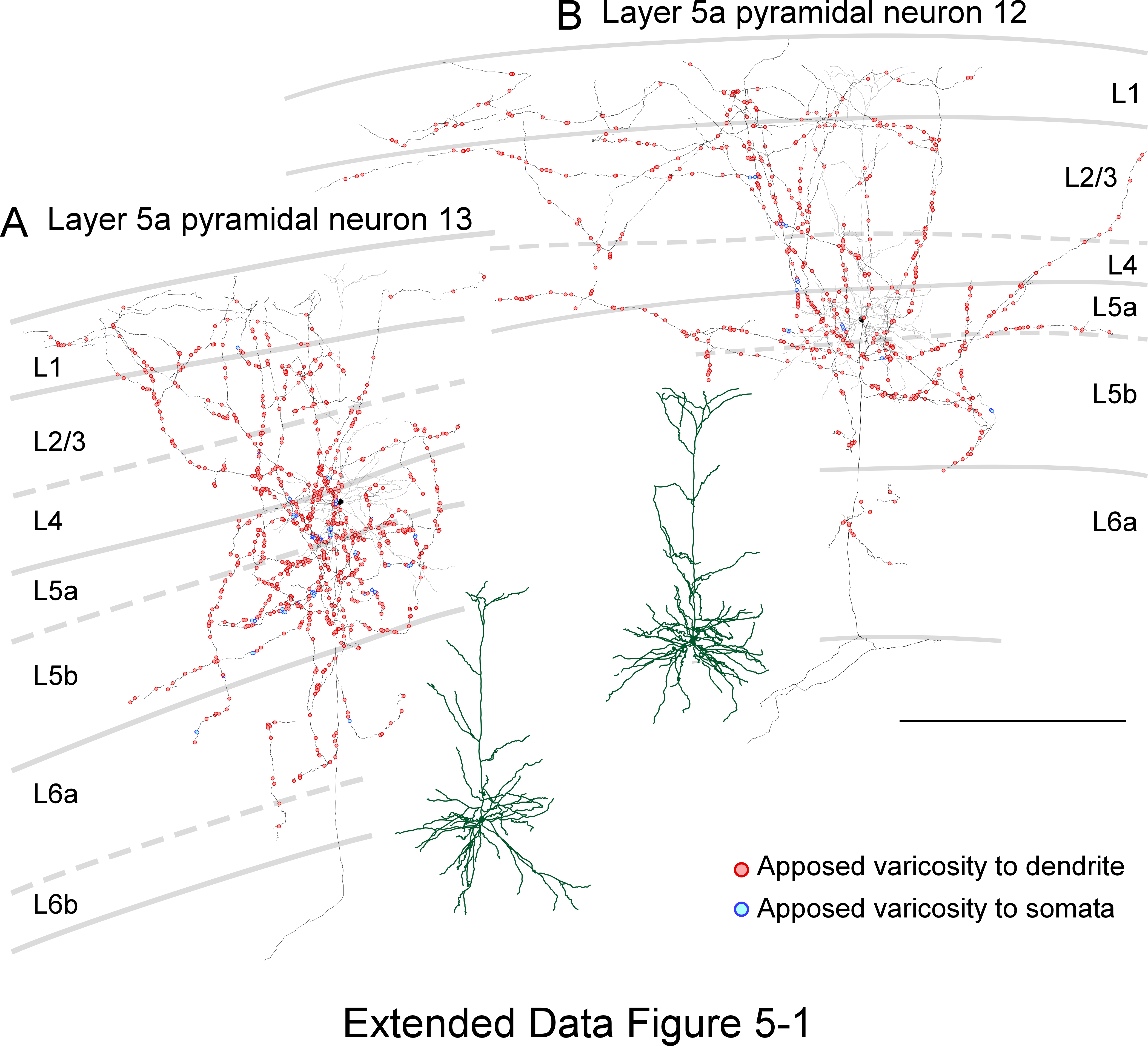

Supplement: Extended Data Figure 5-1 — Distribution of varicosities of layer 5a pyramidal neurons in close appositions to PV neurons. Axons of layer 5a pyramidal neurons were reconstructed two-dimensionally and projected onto the frontal plane. Black lines and filled circles represent the axons and cell bodies of pyramidal neurons, respectively; red and blue circles indicate the axodendritic and axosomatic appositions, respectively. Each apposed varicosity is represented by a red or blue circle. Dark green and gray lines indicate reconstructed dendrites and their actual positions in the cortical layers, respectively. These reconstructed layer 5a pyramidal neurons had less developed apical dendrites than layer 5b neurons. Scale bar: 500 μm. Download Figure 5-1, TIF file. [file enu-eN-NWR-0567-20-s06.tif]

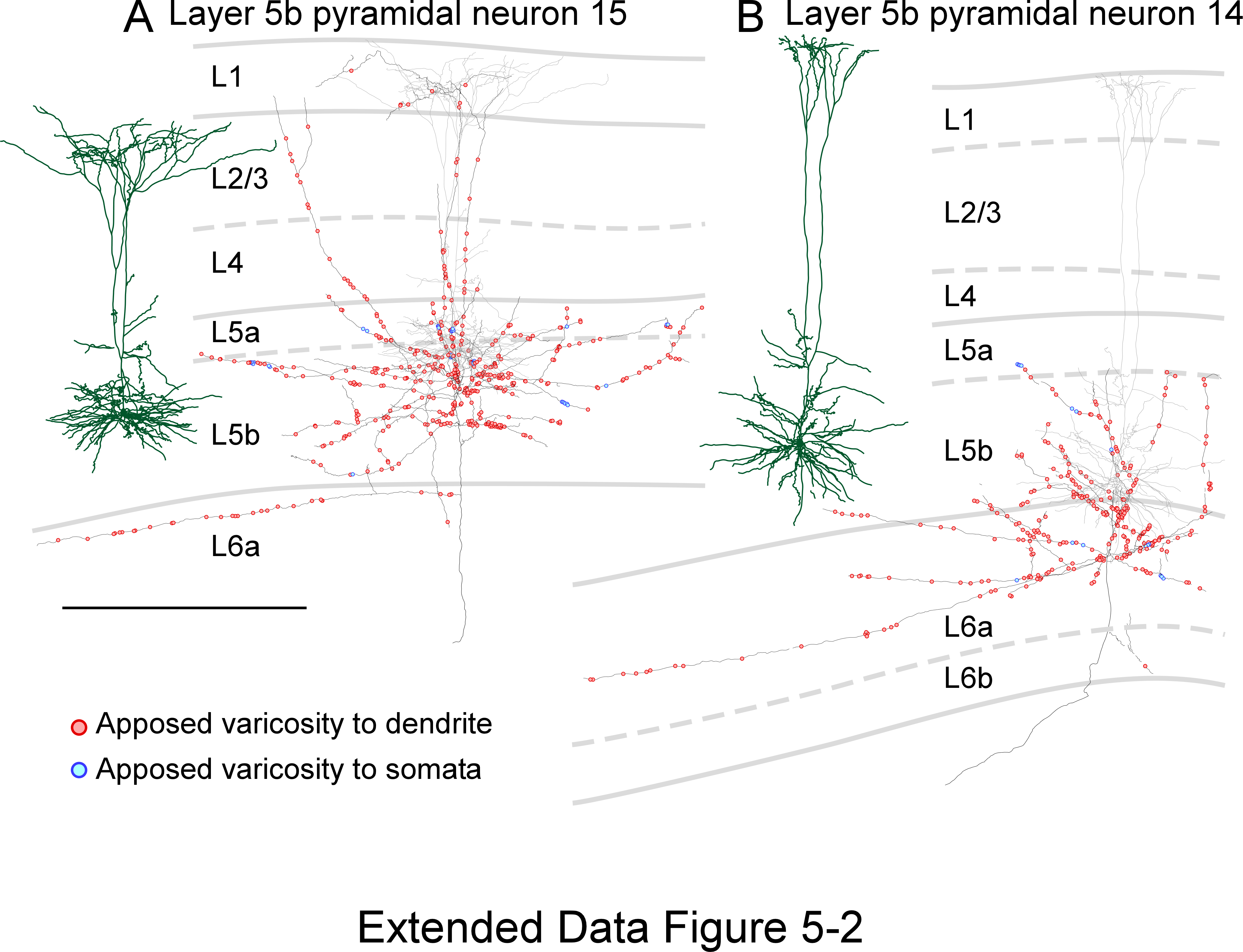

Supplement: Extended Data Figure 5-2 — Distribution of varicosities of layer 5b pyramidal neurons in close appositions to PV neurons. Axons of layer 5b pyramidal neurons were reconstructed two-dimensionally and projected onto the frontal plane. Black lines and filled circles represent axons and cell bodies of pyramidal neurons, respectively; red and blue circles indicate the axodendritic and axosomatic appositions, respectively. Each apposed varicosity is represented by a red or blue circle. Dark green and gray lines indicate reconstructed dendrites and their actual positions in the cortical layers, respectively. These reconstructed layer 5b pyramidal neurons had more abundant apical dendrites than layer 5a neurons. Scale bar: 500 μm. Download Figure 5-2, TIF file. [file enu-eN-NWR-0567-20-s07.tif]

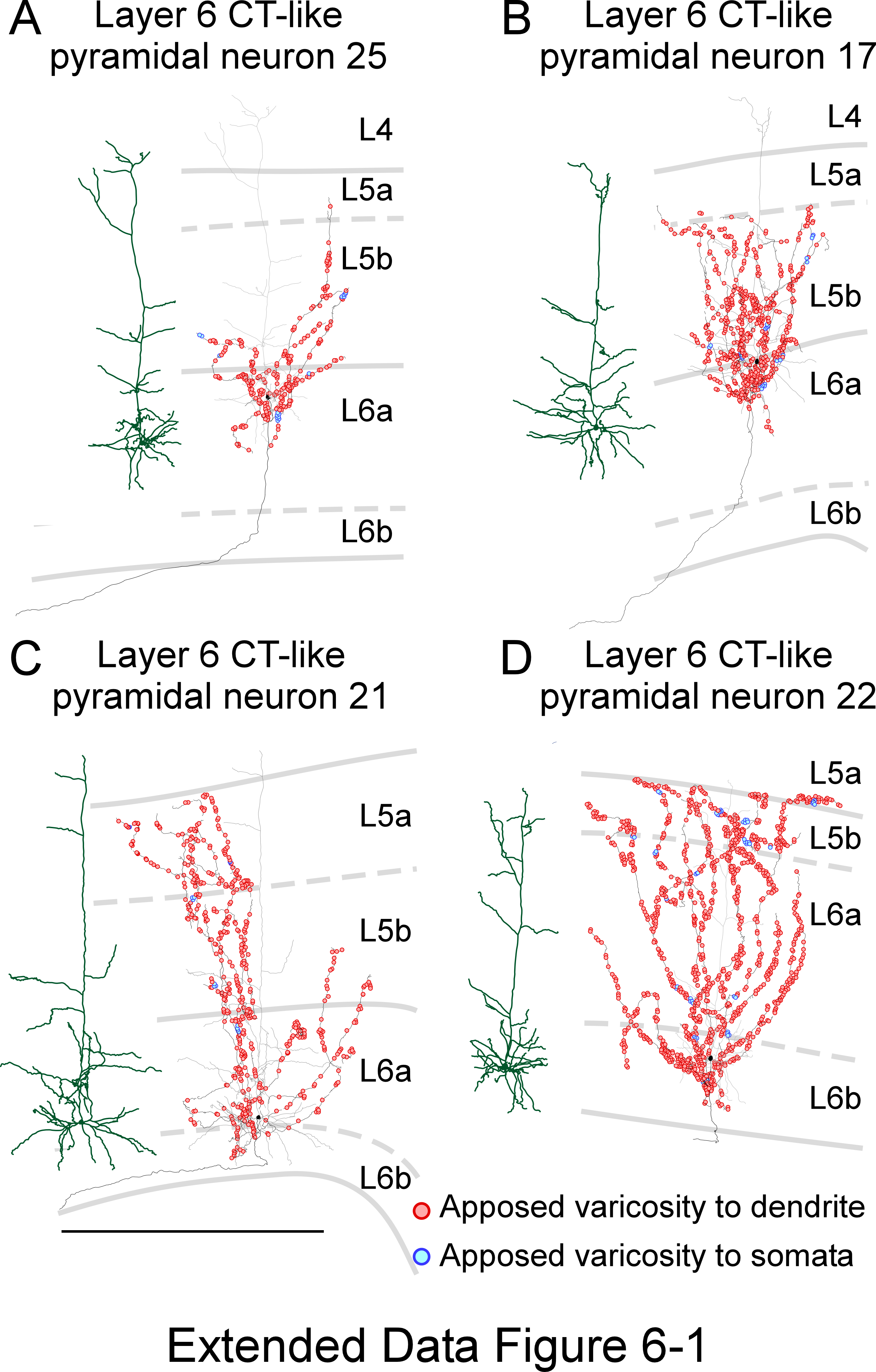

Supplement: Extended Data Figure 6-1 — Distribution of varicosities of layer 6 CT-like pyramidal neurons in close appositions to PV neurons. Axons of layer 6 CT-like pyramidal neurons were reconstructed two-dimensionally and projected onto the frontal plane. Black lines and filled circles represent axons and cell bodies of pyramidal neurons, respectively; red and blue circles indicate the axodendritic and axosomatic appositions, respectively. Each apposed varicosity is represented by a red or blue circle. Dark green and gray lines indicate reconstructed dendrites and their actual positions in the cortical layers, respectively. Neurons 17 and 25 had apical dendrites that terminated in layer 4, and neurons 21 and 22 possessed apical dendrites that terminated in layer 5, suggesting that they were appeared to be Type II and Type I CT-like neurons, respectively. See the text for further details. Scale bar: 500 μm. Download Figure 6-1, TIF file. [file enu-eN-NWR-0567-20-s08.tif]

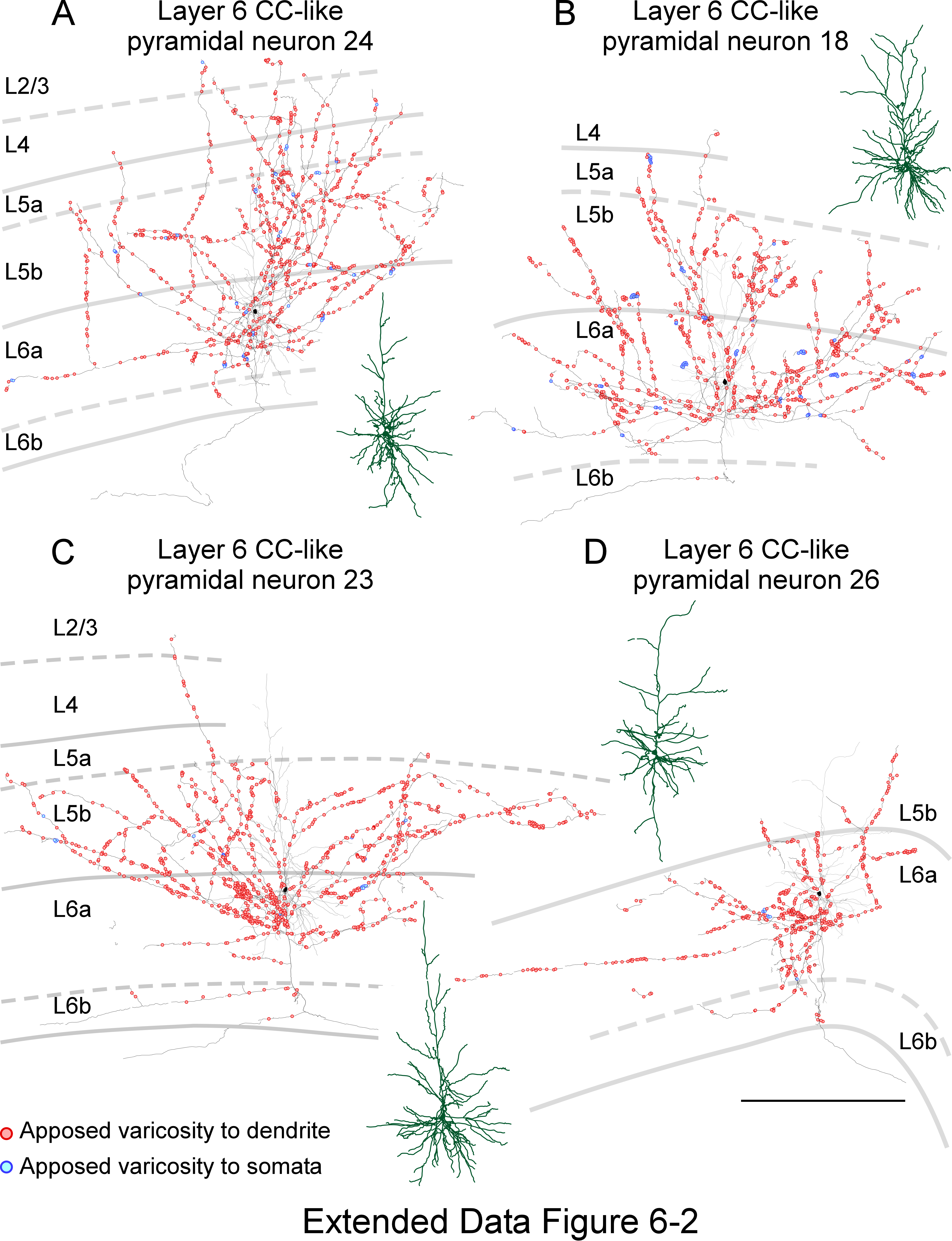

Supplement: Extended Data Figure 6-2 — Distribution of varicosities of layer 6 CC-like pyramidal neurons in close appositions to PV neurons. Axons of layer 6 CC-like pyramidal neurons were reconstructed two-dimensionally and projected onto the frontal plane. Black lines and filled circles represent axons and cell bodies of pyramidal neurons, respectively; red and blue circles indicate the axodendritic and axosomatic appositions, respectively. Each apposed varicosity is represented by a red or blue circle. Dark green and gray lines indicate reconstructed dendrites and their actual positions in the cortical layers, respectively. Scale bar: 500 μm. Download Figure 6-2, TIF file. [file enu-eN-NWR-0567-20-s09.tif]
